# Insights Into the Genetic Architecture of Complex Traits in Napier Grass (*Cenchrus purpureus*) and QTL Regions Governing Forage Biomass Yield, Water Use Efficiency and Feed Quality Traits

**DOI:** 10.3389/fpls.2021.678862

**Published:** 2022-01-07

**Authors:** Meki S. Muktar, Ermias Habte, Abel Teshome, Yilikal Assefa, Alemayehu T. Negawo, Ki-Won Lee, Jiyu Zhang, Chris S. Jones

**Affiliations:** ^1^Feed and Forage Development, International Livestock Research Institute, Addis Ababa, Ethiopia; ^2^Grassland and Forages Division, National Institute of Animal Science, Rural Development Administration, Cheonan, South Korea; ^3^State Key Laboratory of Grassland Agro-Ecosystems, Key Laboratory of Grassland Livestock Industry Innovation, Ministry of Agriculture and Rural Affairs, Engineering Research Center of Grassland Industry, Ministry of Education, College of Pastoral Agriculture Science and Technology, Lanzhou University, Lanzhou, China; ^4^Feed and Forage Development, International Livestock Research Institute, Nairobi, Kenya

**Keywords:** agro-morphological, elephant grass, GWAS, feed quality, anthocyanin, QTL, water-use efficiency

## Abstract

Napier grass is the most important perennial tropical grass native to Sub-Saharan Africa and widely grown in tropical and subtropical regions around the world, primarily as a forage crop for animal feed, but with potential as an energy crop and in a wide range of other areas. Genomic resources have recently been developed for Napier grass that need to be deployed for genetic improvement and molecular dissection of important agro-morphological and feed quality traits. From a diverse set of Napier grass genotypes assembled from two independent collections, a subset of 84 genotypes (although a small population size, the genotypes were selected to best represent the genetic diversity of the collections) were selected and evaluated for 2 years in dry (DS) and wet (WS) seasons under three soil moisture conditions: moderate water stress in DS (DS-MWS); severe water stress in DS (DS-SWS) and, under rainfed (RF) conditions in WS (WS-RF). Data for agro-morphological and feed quality traits, adjusted for the spatial heterogeneity in the experimental blocks, were collected over a 2-year period from 2018 to 2020. A total of 135,706 molecular markers were filtered, after removing markers with missing values >10% and a minor allele frequency (MAF) <5%, from the high-density genome-wide markers generated previously using the genotyping by sequencing (GBS) method of the DArTseq platform. A genome-wide association study (GWAS), using two different mixed linear model algorithms implemented in the GAPIT R package, identified more than 35 QTL regions and markers associated with agronomic, morphological, and water-use efficiency traits. QTL regions governing purple pigmentation and feed quality traits were also identified. The identified markers will be useful in the genetic improvement of Napier grass through the application of marker-assisted selection and for further characterization and map-based cloning of the QTLs.

## Introduction

Improving livestock feeds and forages will play a key role in global food and nutrition security and have the potential to contribute to the strategy of achieving climate-smart agriculture, restoring degraded lands and decreasing greenhouse gas emission intensities (Bryan et al., 2013; Peters et al., 2013; Belay, 2019; Paul et al., 2020). The availability of adequate, high-quality feeds and forages has been a major challenge faced by the livestock sector, especially during the dry season when pasture and crop residues are scarce (Mtengeti et al., 2008; Maleko et al., 2018). To cope with the shortage of feeds during the dry season, many farmers in sub-Saharan Africa (SSA) rely mainly on drought-tolerant perennial grasses, such as Napier grass, that can produce a reasonable amount of feed under limited water availability (Lukuyu et al., 2012; Kabirizi et al., 2015).

Napier grass (*Cenchrus purpureus* (Schumach.) Morrone syn. *Pennisetum purpureum* Schumach.), also called elephant grass, belonging to the poaceae family, is one of the most important perennial tropical C4 grasses (Robert et al., 2010). Napier grass is native to SSA from where it has been distributed to other tropical and subtropical regions around the world, adapting to a wide range of soil and agro-ecological conditions (Negawo et al., 2017). Napier grass has been adapted to areas of North and South America, tropical parts of Asia, Australia, the Middle East, and, the Pacific (Anderson et al., 2008; Negawo et al., 2017). Napier grass is cultivated primarily as a forage crop for animal feed in cut-and-carry feeding systems, it is particularly well-known by smallholder farmers in Eastern and Central Africa (Lukuyu et al., 2012; Kabirizi et al., 2015). Napier grass is known for its high biomass production (up to 78 tons of dry matter per hectare annually), year-round availability under limited irrigation, ability to withstand repeated cuttings when harvested multiple times, resistance to most pests and diseases, ease of establishment and rapid propagation and, fast regrowth capacity (Anderson et al., 2008; Lukuyu et al., 2012; Kabirizi et al., 2015). Napier grass is also used in the push-pull integrated pest management strategy (Khan et al., 2011; Van den Berg and Van Hamburg, 2015), is commonly grown around many crops as a wind and fire break and, is planted in marginal lands and slopes to increase soil fertility and to reduce soil erosion (Kabirizi et al., 2015; Negawo et al., 2017). Recently, reports have shown the potential of Napier grass for biofuel, bioremediation and paper production (Madakadze et al., 2010; Rengsirikul et al., 2013; Tsai and Tsai, 2016; Rocha-Meneses et al., 2020).

Napier grass is an allotetraploid (2n = 4x = 28) with a complex genome (A′A′BB genomes, with A′ genome showing a high degree of homology with the pearl millet A genome) and high genetic diversity, which is mainly attributed to the wide parental diversity and its out-crossing nature (Robert et al., 2010; Dos Reis et al., 2014). Despite its multipurpose use, enormous potential in a wide range of areas and its high genetic diversity, there have been limited efforts to develop varieties with high forage value through breeding and genetic studies. Most of the genetic studies conducted so far were limited to characterizing its genetic diversity using low-density molecular markers (Babu et al., 2009; Azevedo et al., 2012; Wanjala et al., 2013; Kandel et al., 2016; Negawo et al., 2018) that provide a poor representation of the whole-genome information. The generation of high-density genome-wide markers using the genotyping by sequencing (GBS) method permitted the construction of the first high-density genetic map (Paudel et al., 2018) and the first detailed genetic diversity and population structure analysis (Muktar et al., 2019) in Napier grass. The latter study (Muktar et al., 2019) provided a useful insight into the genetic diversity and genome-wide patterns of linkage disequilibrium (LD) in two Napier grass collections, both the material maintained in the International Livestock Research Institute (ILRI) forage genebank and a collection acquired from the Brazilian Agricultural Research Corporation (EMBRAPA) and demonstrated the potential of the collections for further genetic and marker-trait association studies. The most important breakthrough was the recent reports of the first high-quality chromosome scale genome sequences of Napier grass (Zhang et al., 2020; Yan et al., 2021), in which a 1.97 to 2.07 Gb genome was assembled. This chromosome scale genome sequence offers significant opportunities for the dissection of the genetic architectures of complex traits and the development of improved Napier grass varieties.

Molecular tools need to be deployed for Napier grass improvement and the dissection of important agronomic and feed quality traits, for example, by linking sequence polymorphisms with traits using genome-wide association studies (GWAS) and/or linkage mapping. The GWAS technique, which is based on LD, is well established and a potential approach for genetic dissection of complex traits and the identification of quantitative trait loci (QTLs) (Flint-Garcia et al., 2003; Huang and Han, 2014). DNA markers identified by GWAS for agronomic traits have been successfully exploited in several crop plants, for marker-assisted selection (MAS), gene cloning, trait improvement and designing an effective breeding strategy (Burridge et al., 2017; Guo et al., 2018; Sanchez et al., 2018; Jaiswal et al., 2019a). Unlike classical linkage mapping that uses a population of the progeny of a biparental cross, GWAS is performed on a diverse collection of unrelated genotypes (Flint-Garcia et al., 2003; Huang and Han, 2014) and hence this technique is ideally suited to the study of genebank collections (McCouch et al., 2012). To date, only two GWAS studies on Napier grass have been reported (Rocha et al., 2019; Habte et al., 2020), in which markers associated with high biomass yield, metabolizable energy and biomass digestibility were detected. However, these studies had a limitation on either marker density (a total of 111 alleles from 18 SSR markers on 90 Napier grass genotypes were used for GWAS in Rocha et al., 2019) or population size (a total of 45 Napier grass genotypes were used in Habte et al., 2020).

Here, we report on the first marker-trait association and QTL identification in Napier grass in a controlled and replicated field trial, with repeated trait measurements, high-density genome-wide markers and different statistical approaches. Selected Napier grass genotypes have been evaluated in a field in dry (DS) and wet (WS) season conditions. Morphological, agronomic, water-use efficiency and feed quality traits were collected over 2 years from 2018 to 2020, the trait values were subjected to a spatial analysis (Rodríguez-Álvarez et al., 2018) and adjusted for the spatial heterogeneity of the experimental blocks. GWAS was employed using the adjusted phenotypic data and high-density genome-wide markers generated previously (Muktar et al., 2019), with the objective of dissecting the genetic architecture of complex traits in Napier grass and identifying markers and QTL regions associated with forage-biomass yield, water-use efficiency and feed quality traits.

## Materials and Methods

### Selection of High-Density Genome-Wide Markers

The Napier grass collections have been genotyped by the Diversity Array Technology^1^ using the DArTseq platform as described previously (Muktar et al., 2019). High density genome-wide SilicoDArT (presence/absence), and SNP markers were called following the DArTseq protocol (Kilian et al., 2012).

From the high-density genome-wide markers generated, a total of 135,706 markers (90,498 silicoDArTs and 45,208 SNPs) were filtered after removing markers with missing values > 10% and a minor allele frequency (MAF) < 5%. The markers with 10% missing values were imputed using the missForest R package (Stekhoven and Bühlmann, 2012), with maxiter set to 5 and ntree to 100 and all other parameters set to default values. The imputation was run on one assembled chromosome (AC) at a time as the run time of the software could not accommodate taking the entire genome at once. The short sequence reads corresponding to the markers were aligned to the recently reported Napier grass genome (Yan et al., 2021) using the bwa mem sequence aligner v0.7.17 (Li and Durbin, 2009) and the marker density and distribution across the fourteen ACs of the Napier grass genome was visualized using the synbreed R-package (Wimmer et al., 2012). The sequences were annotated using the genomic information resources of *Cenchrus americanus* and *Setaria italica* in the GenBank NCBI blastx tool^2^ by the technique of reciprocal blastx, as described previously (Muktar et al., 2019). The BLAST results (*C. americanus* to *S. italica* and *S. italica* to *C. americanus*) with best scores (a BLASTP Expect value of less than 10) were joined using the “subject” and “query” fields and reciprocal blast best hits were produced. The annotation information for *S. italica* and *P. glaucum* was extracted using UniProt (free-text gene function and Gene Ontology annotations) and merged with the reciprocal blast best hits, which was in turn matched with the markers based on their genomic position.

### Marker Data Analysis, Linkage Disequilibrium and LD-Decay Analysis

The missing percentage data, minor allele frequency (MAF) per marker and per genotype, and the polymorphic information content (PIC) were calculated in R statistical software^3^ as described previously (Muktar et al., 2019). Pair-wise linkage disequilibrium (LD) between pairs of SilicoDArT markers (SilicoDArTs were selected as the DArTseq technology produces more precise genomic position information for this marker than for SNPs) with a known genomic location on the Napier grass genome (Yan et al., 2021) was estimated based on the correlation coefficient (*r*^2^) calculated using PLINK v1.09 (Purcell et al., 2007). The LD was estimated only for markers located on the same chromosome, then the *r*^2^ values from all chromosomes were pooled and plotted against the physical distance between markers to estimate the average rate of LD decay across the whole genome, as described in Muktar et al. (2019).

### Field Planting, Drought Stress Application and Trait Measurements

A total of 84 genetically diverse genotypes (the origin and diversity of the genotypes were well documented in Muktar et al., 2019) were planted in June 2017 during the main rainy season (mid-June to mid-September) at the Bishoftu field site, Ethiopia, which is located at 008_4702000 N and 038_5901500 E, at an altitude of 1890 m above sea level, about 48 km southeast of Addis Ababa, and has an Alfisol type of soil. The genotypes were arranged in a partially replicated (p-rep) design in four blocks where a selected 12 genotypes (14% of the population) were duplicated in each block as checks to control for soil heterogeneity as described previously (Muktar et al., 2019). Six plants per accession were planted in a single row, with 750 mm spacing between plants and between rows. A border plant (acc. ILRI_14984) was planted around each block to reduce border effects. A trench, approximately 1m deep, was dug between blocks to avoid seepage of water from one block to another. During the dry season (DS), two blocks were irrigated to a volumetric soil water content (VWC) of approximately 20% (now onwards called moderate water stress, MWS) and the other two blocks were irrigated with a reduced amount of water, which corresponds to a VWC of about 10% (now onwards called severe water stress, SWS) (Supplementary Figure 1). There was no irrigation in the wet season (WS) as all plants were maintained under rainfed (RF) conditions (approximately 30% VWC) (Supplementary Figures 1, 2). Approximately 3 months after establishment, the plants were clean cut to a standard height of 50 mm above ground, subsequently, harvesting and data collections were conducted following every 8 weeks of regrowth. The first four harvests were considered as an establishment period and data from the fifth harvest onwards were used in the data analysis. Six harvests per year, 12 harvests overall, were conducted across a 2 years period. Data of morphological, agronomic, and feed quality traits were collected from three randomly selected plants per row in each of the three soil moisture conditions, as described in Habte et al. (2020). The agro-morphological traits collected were plant height (PH) in centimeter (cm), leaf length (LL) in cm, leaf width (LW) in millimeter (mm), stem thickness (ST) in mm, tiller number (TN) count, internode length (IL) in cm, total fresh weight (TFW) in t/ha, total dry weight (TDW) t/ha, leaf-stem-ratio (LSR), and water use efficiency (WUE) in g/l (Dry matter produced per liter of water). For the feed quality traits, Acid detergent fiber (ADF) in %, acid detergent lignin (ADL) in %, crude protein (CP) in %, dry matter (DM), *in vitro* organic matter digestibility (IVOMD), metabolizable energy (Me) in J/KgDM, neutral detergent fiber (NDF) in %, organic matter (OM) in %, were collected.

### Phenotypic Data Analysis and Correction for Spatial Variation

The phenotypic value of each trait was adjusted according to the spatial variation across the experimental field using the SpATS R-package (Rodríguez-Álvarez et al., 2018) in R (R Core Team, 2021) statistical software. The analysis was performed individually for each of the three soil moisture conditions. In this study, plots were laid out in 24 row by 4 column grids in each block, thus rows and columns were used as random factors. In addition, the multi-harvests, soil moisture data and soil nutrient parameters (Acid = soil acidity, AvaP = available phosphorus, K = available potassium, OM = organic matter, and CEC = cation exchange capacity) were included in the mixed model as fixed covariates. Thus, the following SpATS mixed model for each treatment condition was fitted;


(1) y=Xβ+f(u,v)+ZCr+rZCc+c↋

where y is phenotypic observation; β is a vector of random genetic (genotype) effects, with X as the corresponding design matrix; f (u,v) are vectors of row and column random effects; Z_r_ and Z_c_ are vectors of fixed effects of replications, multi-harvests, soil-moisture, and soil-nutrient parameters, with C_r_ and C_c_ as the corresponding random effect coefficients for the rows and columns, respectively; ε is the random residual error (Rodríguez-Álvarez et al., 2018).

The pairwise correlation between all possible trait-pairs was assessed using the R function “*cor_pmat*” in the package ggcorrplot in R (R Core Team, 2021) and visualization of the correlation matrices was undertaken using the “*ggcorrplot*” function. Effective dimension (ED), which is a measure of the complexity of the model components, and broad-sense heritability based on the adjusted data were generated by the R functions “*summary*” and “*getHeritability*,” respectively, in the SpATS R-package (Rodríguez-Álvarez et al., 2018). For comparison purposes, broad-sense heritabilities were also analyzed for the unadjusted data using the “*mmer*” function in the sommer R package (Covarrubias-Pazaran, 2016), using the formula;


(2) $$H^{2}=\frac{\sigma_{g}^{2}}{\begin{array}{ccc} \sigma_{g}^{2}+ & \frac{\sigma_{\text{gh}}^{2}}{n}+\frac{\sigma_{\text{gr}}^{2}}{r}+ & \frac{\sigma_{e}^{2}}{nr} \end{array}}$$

Where *H*^2^ is broad-sense heritability; $\sigma_{g}^{2}$ is the genotype variance; $\sigma_{\text{gh}}^{2}$ is the variance due to genotype by harvest interaction; $\sigma_{\text{gr}}^{2}$ is the variance due to genotype by block interaction; *n* is the number of harvests; *r* is the number of blocks (replications) per season (equal to two for dry and four for wet seasons); and $\sigma_{e}^{2}$ is the error variance.

The normality of the data for each trait was tested by drawing normal plots in a histogram by using the “*hist*” function in R (R Core Team, 2021).

### Marker Trait Association Analysis

Marker-trait association analysis was carried out using 83 Napier grass genotypes (one genotype was excluded because of high missing values for the marker data) that had been genotyped and phenotyped (adjusted for spatial variation as described above).

For the agro-morphological and feed quality quantitative traits, the analysis was performed using the Bayesian-information and Linkage-disequilibrium Iteratively Nested Keyway (BLINK.R) (Huang et al., 2018) and multiple-locus mixed linear model (MLMM) (Segura et al., 2012) algorithms implemented in Genomic Association and Prediction Integrated Tool version 3 (GAPIT3) (Wang and Zhang, 2021). The BLINK.R model is based on linkage disequilibrium (LD) and eliminates confounding issues arising due to population structure, kinship, multiple testing correction, etc. The first three to five components identified through principal component analysis (PCA) using the Adegenet R package (Jombart, 2008) were included as covariates in the model. The number of components increased from three to five until the quantile-quantile (Q-Q) plot shows a similar distribution between observed and expected *P*-values along a solid diagonal line except for a sharp curve of the observed *P*-value at the end of the line, which represents a true association. The MLMM model was based on a PC+K that represent population structure and relatedness, respectively. This model uses stepwise regression to introduce significant markers as cofactors in each step of the model, thereby excluding collinear markers in strong LD with the same locus (Segura et al., 2012). To control for type I errors due to multiple testing, the *p*-values were adjusted following a false discovery rate (FDR) correction procedure (Benjamini and Hochberg, 1995). Markers detected by both models were claimed to be associated.

In addition, marker-trait association analysis was carried out for the qualitative trait, purple pigmentation, by computing the non-parametric univariate Fisher’s exact test (Warner, 2013) using the Adegenet R package (Jombart, 2008). Out of the diverse set of Napier grass genotypes acquired from EMBRAPA (Negawo et al., 2018; Muktar et al., 2019), seven had purple-colored leaves, midribs, petioles and stems (Supplementary Figure 3). The plant colors were qualitatively scored with a “1” for the seven purple-colored genotypes and “0” for 98 green-colored genotypes (additional genotypes that were not phenotyped in the field were included in this case, assuming that will increase the power of the QTL identification). A threshold level at *P-*value < 1.00E-07 (> the −log10 of 7) was used to claim an association.

The genomic map position of associated markers and their co-localization was estimated based on the sequence length of each of the 14-assembled chromosomes (AC) (Yan et al., 2021) and a physical map was constructed at a 1Mbp scale.

## Results

### Spatially Corrected Phenotype Data, Distribution, and Correlation of Traits

**FIGURE 1:**
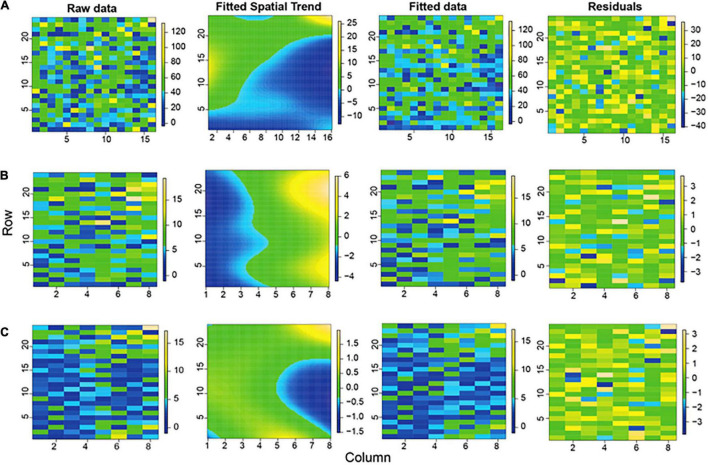
Graphical representations of the raw data, fitted spatial trend, fitted data, and residuals for the biomass trait (total fresh weight, TFW) of the wet season under rainfed (WS-RF) **(A)**, under moderate water stress in dry season (DS-MWS) **(B)**, and severe water stress in dry season (DS-SWS) **(C)** conditions.

The phenotypic measurements of the agronomic, morphological, and feed quality traits were collected on a genetically diverse set of 84 Napier grass genotypes evaluated over a 2-year period under three soil moisture conditions in the wet (WS) and dry (DS) seasons. The average soil volumetric water content (VWC) of each of six harvests under the three soil moisture conditions (WS-RF, DS-MWS, and DS-SWS) is shown in Supplementary Figure 1. The phenotypic values were corrected for spatial heterogeneity across rows and columns of the experimental blocks, as well as for the heterogeneity in soil moisture content (SM) and soil nutrient parameters. The depicted spatial trend representing the estimated heterogeneity across rows and columns of the experimental blocks for each treatment is shown in Figure 1.

The model captured more spatial heterogeneity toward the columns than the rows as suggested by effective dimension (ED) (Table 1), which is a measure of the complexity of the SpATS model (Rodríguez-Álvarez et al., 2018). This column-wise spatial heterogeneity was more pronounced in the WS (four blocks) than in the DS (two blocks in each treatment). As shown by the spatial trend (Figures 1A,C), plants grown in the middle part of block 4 performed worse compared to the plants in the other blocks which was consistent with visual observations. In the DS, of the two blocks under MWS conditions, plants in block 3 performed better than in block 1 (Figure 1B).

**TABLE 1:** Estimated effective dimension (ED) associated with the spatial trend of the row (R), column (C), and genetic (G) random factors for each trait.

Trait	WS-RF					DS-MWS					DS-SWS				
	ED (C)	ED (R)	ED (G)	H^2^_g_	H^2^_gf_	ED (C)	ED (R)	ED (G)	H^2^_g_	H^2^_gf_	ED (C)	ED (R)	ED (G)	H^2^_g_	H^2^_gf_
PH	10.5	0.0	73.9	0.80	0.89	0.8	1.3	71.1	0.59	0.86	0.0	0.0	61.5	0.31	0.74
LL	10.1	0.0	74.9	0.85	0.90	NA	NA	NA	NA	NA	2.6	0.0	57.1	0.41	0.69
LW	8.7	5.6	75.5	0.82	0.91	1.1	2.3	73.6	0.60	0.89	0.0	0.0	61.1	0.36	0.74
ST	4.3	0.0	24.7	0.19	0.30	1.9	0.0	46.9	0.49	0.57	2.5	0.0	45.6	0.40	0.56
TN	0.0	6.9	71.7	0.79	0.86	0.0	8.4	71.1	0.89	0.86	0.0	0.8	67.7	0.81	0.82
IL	8.1	0.1	51.5	0.29	0.62	NA	NA	NA	NA	NA	NA	NA	NA	NA	NA
LSR	0.2	0.1	10.4	0.06	0.12	NA	NA	NA	NA	NA	NA	NA	NA	NA	NA
TFW	5.0	0.1	69.2	0.78	0.83	1.8	0.2	68.2	0.78	0.82	1.0	0.0	58.7	0.66	0.71
TDW	0.1	0.0	58.4	0.51	0.70	2.5	0.0	66.2	0.72	0.80	0.4	0.0	58.9	0.69	0.71
WUE	NA	NA	NA	NA	NA	2.7	0.0	65.2	0.63	0.79	2.5	0.0	58.6	0.63	0.71
ADF	11.3	0.0	66.1	0.52	0.70	0.0	0.9	59.4	0.49	0.62	0.0	8.1	51.0	0.38	0.51
ADL	9.1	0.0	60.1	0.40	0.62	0.0	0.1	72.1	0.66	0.77	0.0	6.2	59.8	0.49	0.62
CP	7.4	1.0	44.0	0.23	0.43	2.1	0.0	61.0	0.59	0.64	0.0	3.9	53.7	0.29	0.55
ash	7.0	0.1	69.4	0.63	0.74	2.7	3.9	68.6	0.73	0.73	4.0	6.4	48.9	0.53	0.59
DM	8.2	0.0	48.8	0.28	0.49	2.0	2.1	70.9	0.70	0.75	0.0	0.0	62.1	0.48	0.65
IVOMD	8.2	0.0	48.5	0.27	0.48	1.1	0.0	58.3	0.52	0.60	0.0	2.0	47.9	0.23	0.48
Me	10.9	0.0	38.9	0.22	0.37	0.3	0.6	53.1	0.51	0.47	0.1	6.0	32.5	0.24	0.39
NDF	4.5	0.0	62.2	0.40	0.65	0.0	0.1	69.5	0.70	0.74	2.7	0.0	63.7	0.59	0.67
OM	6.5	0.0	67.2	0.60	0.71	2.7	3.9	68.6	0.73	0.73	4.0	0.0	52.6	0.50	0.53

*Broad-sense heritability for unfitted (H^2^g) and fitted (H^2^gf) data for the wet season under rainfed (WS-RF), dry-season under moderate water stress (DS-MWS), and dry season under severe water stress (DS-SWS) conditions are shown. PH, plant height; LL, leaf length; LW, leaf width; ST, stem thickness; TN, tiller number; IL, internode length; LSR, leaf stem ratio; TFW, total fresh weight; TDW, total dry weight; WUE, water-use efficiency; ADF, acid detergent fiber; ADL, acid detergent lignin; CP, crude protein; DM, dry matter; IVOMD, in vitro organic matter digestibility; Me, metabolizable energy; NDF, neutral detergent fiber; OM, organic matter.*

Correcting the phenotypic values according to the spatial heterogeneity improved the precision of the heritability estimates and mostly increased the heritability value of the traits (Table 1), indicating the importance of the spatial analysis to determine the genetic and environmental effects on the phenotypic response and to reduce the environmental effects and errors. The heritability of traits ranged from 0.12 to 0.91 in the WS, 0.57 to 0.89 in the DS under MWS and 0.39 to 0.82 in the DS under SWS conditions. Traits LW, LL, PH, TN, ash, TFW, OM, and ADF had the highest heritability followed by NDF, ADL, TDW, and WUE, while ST, Me, and IL had low to medium heritabilities. LSR had the lowest heritability, which indicates that the variation in the trait was mainly due to environmental factors. Therefore, the LSR trait was excluded from the GWAS analysis. With LSR excluded, heritability generally decreased in the DS, particularly under the SWS condition (Table 1).

In the correlation analysis, TFW, TDW, and WUE showed a strong positive correlation (>0.97), while LSR was strongly negatively correlated with all the traits except with IL. TN had a weak negative correlation with IL and LW, while IL showed a weak to moderate negative correlation with LW, TN, PH, TFW, and TDW (Supplementary Figure 4). The phenotypic values of all the traits followed a normal distribution, only the distribution of LL under the WS-RF and DS-SWS conditions and ST and LW in the WS-RF were slightly skewed to the left (Supplementary Figure 5).

From the feed quality traits, IVOMD and Me and IVOMD and CP showed a strong positive correlation (>0.90) followed by a strong correlation between Me and CP, ADL and ADF and NDF and OM. Conversely, OM and ash showed a very strong negative correlation (>−0.97), followed by a strong negative correlation between IVOMD and ADF, Me and ADF, NDF and ash, and, ADF and CP (Supplementary Figure 6). The values of all the traits showed a normal distribution (Supplementary Figure 7).

There were no strong positive/negative correlations between traits from the agro-morphological and feed quality traits. However, most of the agro-morphological traits, except TN, were reasonably positively correlated (0.30 to 0.60) with the traits linked with fiber components (ADF and NDF) and negatively with traits positively affecting the feed nutritional quality (CP, IVOMD, and Me).

### Genome-Wide Distribution and Density of Markers on the Napier Grass Genome

**FIGURE 2:**
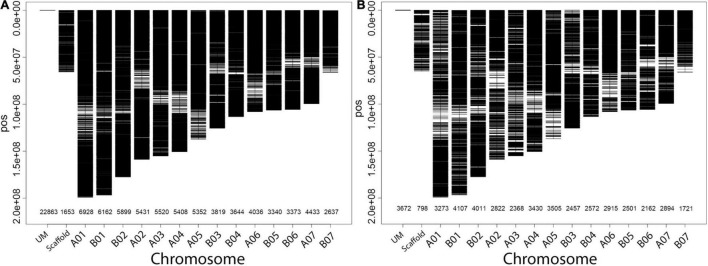
Distribution and density of silicoDArTs **(A)** and SNPs **(B)** on the fourteen assembled chromosomes (AC) of the Napier grass genome. The number of markers mapped per AC is shown on the *x*-axis. The markers that were not mapped are indicated by a UM and those markers that were mapped onto different scaffolds are indicated by Scaffold.

Out of more than 200,000 high density genome-wide SilicoDArT and SNP markers (Muktar et al., 2019) generated on the Napier grass collections, a total of 135,706 (90,498 silicoDArTs and 45,208 SNPs) markers were mapped on to the Napier grass genome (Yan et al., 2021) and their distribution across the fourteen assembled chromosomes (AC) is shown in Figure 2. Approximately 80% of the markers were mapped on the genome. The highest number of markers mapped onto A01 and B01, while the lowest number mapped onto B07 (Figure 2), with a strong correlation (>0.94) between number of markers and chromosome size (Table 2). Polymorphic information content (PIC) values of the markers ranged from 0.08 to 0.38 with an average of 0.27 and more than 63% of the markers had a PIC value above 0.25.

**TABLE 2:** Density and distribution of markers, proportion of pairwise marker in linkage disequilibrium (LD) and the average LD-decay distance in each of the 14 assembled chromosomes (AC).

AC	AC-size*^i^* (Mbp)	Number of markers	markers density (per Mbp)	Proportion of marker-pairs in LD (*r*^2^ ≥ 0.2)	Proportion of marker-pairs in LD (*r*^2^ ≥ 0.7)*^ii^*	LD-decay distance (kbp)
A01	199.06	6928	34.80	15.98	2.58	7.58
B01	196.76	6162	31.32	13.61	1.78	4.61
B02	177.74	5899	33.19	13.01	1.58	2.68
A02	158.81	5431	34.20	14.45	2.37	5.47
A03	155.16	5520	35.58	14.38	2.37	5.79
A04	150.59	5408	35.91	13.43	1.73	3.85
A05	137.44	5352	38.94	13.19	1.78	2.31
B03	125.68	3819	30.39	13.89	1.68	3.75
B04	113.33	3644	32.15	12.35	1.48	2.48
A06	108.24	4036	37.29	13.45	1.60	2.23
B05	106.42	3340	31.39	12.04	1.37	1.77
B06	106.01	3373	31.82	12.45	1.80	2.14
A07	99.75	4433	44.44	12.39	1.51	1.95
B07	66.05	2637	39.93	12.46	1.80	1.60

*^i^Yan et al. (2021); r^2^ = 0.7 is the default value to determine LD block by the BLINK.R package (Huang et al., 2018).*

### Estimated Linkage Disequilibrium and LD-Decay on the Napier Grass Genome

**FIGURE 3:**
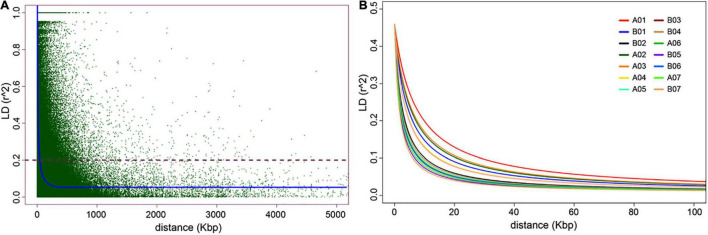
Genome-wide linkage disequilibrium (LD) decay plots. In **(A)**, the average genome-wide LD-decay against the genomic distance (kbp). In **(B)**, the LD-decay per assembled chromosome (AC).

A total of 65,982 genome-wide silicoDArT markers, filtered based on missing values (<10%), MAF (>5%) and known genomic position, were used to estimate linkage disequilibrium (LD) and LD-decay across the Napier grass genome. LD was analyzed between pairs of SilicoDArT markers from the same AC as described previously (Muktar et al., 2019). The magnitude of *r*^2^ (square of the correlation coefficient between two markers) decreased rapidly with an increase in physical genomic distance between markers and reached a value of 0.2 at 3.48 kbp (Figure 3), which is similar to the previous estimation (2.54 kbp) that was based on the pearl millet genome (Muktar et al., 2019). The LD-decay distance varied across ACs and the slowest LD-decay was observed for A01 (7.58 kbp), while the LD decayed rapidly in B05 (1.77 kbp) and B07 (1.60 kbp). LD and LD-decay distance, and the number and density of markers used in the analysis for each AC is shown in Table 2. The proportion of pairwise *r*^2^ values ≥ 0.2 (markers in LD) and ≥ 0.7 (markers in strong LD) per AC ranged from 12.04% in B05 to 15.98% in A01 and 1.37% in B05 to 2.58% in A01, respectively. The LD *r*^2^ ≥ 0.7 was the default value used to determine LD blocks by the model implemented in the BLINK.R package in GWAS analysis (Huang et al., 2018).

### Markers Associated With Quantitative Trait Loci Governing Agro-Morphological Traits

**FIGURE 4:**
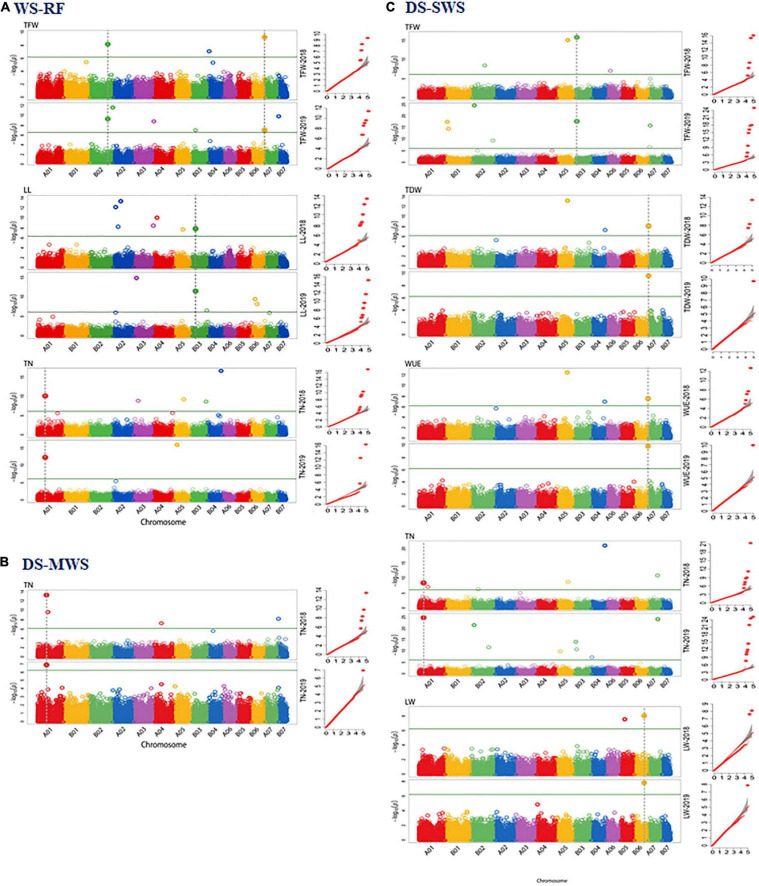
Manhattan and quantile-quantile (Q-Q) plots of markers that showed overlapping associations in 2 years data of the agronomic and morphological traits in wet season under rainfed (WS-RF) **(A)**, dry season under moderate water stress (DS-MWS) **(B)**, and dry season under severe water stress (DS-SWS) **(C)** conditions. Markers are on the *x*-axis per the 14-assembled chromosomes (AC). The –log10 of *P* values are plotted on the *y*-axis. The traits are shown at the left, total fresh weight (TFW), total dry weight (TDW), water-use efficiency (WUE), leaf length (LL), leaf width (LW), and tiller number (TN).

A genome-wide association study (GWAS) was employed using two different mixed linear models implemented in the R package GAPIT3 (Wang and Zhang, 2021). The spatially corrected phenotype values of the agronomic and morphological traits measured in the 83 Napier grass genotypes and the highly polymorphic genome-wide DArTseq markers were used. By using the BLINK.R and MLMM models in GAPIT3, more than 35 markers associated with the agronomic and morphological traits under the three-soil moisture conditions (WS-RF, DS-MWS, and DS-SWS) were identified (Table 3, Figure 4; and Supplementary Figure 8). Markers associated per trait represent different QTLs as the markers are not in LD and therefore are independent of each other.

**TABLE 3:** Markers significantly associated with agro-morphological traits, their genomic positions, contrasting alleles, and minor allele frequency.

Soil moisture condition	Trait	Marker	AC	Pos	allele	MAF	BLINK-model *P*-value			MLMM-model *P*-value		
							2018	2019	Combined	2018	2019	Combined
WS-RF	TFW	IG100206033| F| 0-67:A > G-67:A > G	B02	138389691	A/G	0.46	5.65E-09	8.15E-10	3.65E-12	1.23E-05	NA	8.20E-06
		IG8170757| F| 0-17:C > G-17:C > G	B02	176171258	C/G	0.30	NA	3.63E-12	1.25E-14	NA	NA	1.71E-05
		IG100292170| F| 0-44:C > T-44:C > T	B06	99625821	C/T	0.12	4.63E-10	1.74E-07	6.67E-07	1.07E-05	NA	5.04E-05
	PH	IG100028603	B01	173322345	0/1	0.43	1.75E-05	NA	8.29E-20	9.31E-09	NA	2.25E-26
	LL	IG100007430	B03	27169784	0/1	0.10	1.60E-08	1.94E-12	5.36E-14	2.89E-11	1.27E-23	2.89E-30
	LW	IG100007430	B03	27169784	0/1	0.10	2.46E-14	NA	5.17E-10	5.31E-13	NA	2.21E-19
	TN	IG100292280| F| 0-64:T > G-64:T > G	A01	58504312	T/G	0.06	4.77E-11	2.50E-13	5.38E-09	2.27E-14	1.58E-10	9.48E-12
		IG100028141	A05	28279352	0/1	0.30	NA	5.03E-17	2.27E-09	NA	1.07E-08	3.02E-12
	IL	IG100339862	B05	9736642	0/1	0.24	NA	NA	1.32E-10	NA	NA	6.24E-08
	ST	IG100222553| F| 0-48:C > T-48:C > T	B02	17106838	C/T	0.18	NA	NA	7.66E-10	NA	NA	7.23E-24
		IG100007430	B03	27169784	0/1	0.10	NA	NA	5.38E-30	NA	5.05E-17	1.08E-31

DS-MWS	PH	D23576564	B01	179072922	0/1	0.11	6.71E-05	NA	2.22E-09	5.87E-07	NA	2.39E-06
		IG100244029	A07	42941985	0/1	0.24	NA	NA	7.80E-07	NA	NA	2.69E-09
	LW	IG100337563	A01	179359778	0/1	0.35	NA	NA	9.52E-19	NA	NA	2.21E-20
		IG100202450	B02	80180478	0/1	0.12	NA	NA	6.63E-07	NA	NA	9.23E-09
	TN	IG100008828	A01	67451249	0/1	0.18	1.91E-14	1.15E-07	4.37E-12	3.26E-09	1.15E-07	2.32E-20
		IG100252208	A01	78125692	0/1	0.07	1.70E-10	NA	5.15E-11	8.15E-06	NA	NA
		D23632684	A04	55594226	0/1	0.24	4.12E-08	NA	3.17E-08	4.12E-08	NA	6.90E-17

DS-SWS	TFW	IG100011654	B02	97600432	0/1	0.17	2.20E-09	NA	9.46E-19	7.03E-05	NA	5.61E-11
		D23612801	A02	132204639	0/1	0.29	4.56E-07	NA	2.28E-10	6.39E-05	NA	2.26E-07
		IG100331826	B03	10779294	0/1	0.30	7.76E-17	9.24E-19	2.19E-07	3.18E-07	4.42E-14	1.65E-11
	TDW	IG100288904| F| 0-22:C > G-22:C > G	B02	72599462	C/G	0.20	NA	1.56E-03	2.37E-06	NA	8.52E-09	1.10E-11
		IG100292170| F| 0-44:C > T-44:C > T	B06	99625821	C/T	0.12	5.97E-09	2.04E-10	3.46E-06	NA	1.32E-05	5.48E-05
		IG100044163	A05	80439022	0/1	0.34	3.84E-14	NA	NA	NA	1.11E-10	NA
	WUE	IG100288904| F| 0-22:C > G-22:C > G	B02	72599462	C/G	0.20	NA	2.99E-03	4.15E-14	NA	NA	4.56E-09
		IG100044163	A05	80439022	0/1	0.34	1.95E-13	NA	NA	3.76E-11	NA	1.37E-07
		IG100292170| F| 0-44:C > T-44:C > T	B06	99625821	C/T	0.12	2.02E-08	1.05E-10	4.44E-16	NA	1.88E-05	2.53E-13
	PH	IG100218301	A02	46585403	0/1	0.22	NA	NA	2.02E-08	NA	NA	1.64E-14
		IG100109748| F| 0-53:C > T-53:C > T	B03	81852583	C/T	0.13	NA	NA	8.36E-08	NA	NA	3.29E-12
		D23600991| F| 0-6:G > A-6:G > A	B05	34869947	G/A	0.28	1.73E-12	NA	1.51E-06	7.70E-05	NA	NA
		IG18095269| F| 0-20:C > T-20:C > T	A07	89214569	C/T	0.28	3.02E-08	NA	2.11E-14	NA	NA	8.61E-14
	LW	IG100023744	B06	71606589	0/1	0.45	7.97E-09	1.36E-08	1.20E-16	NA	3.52E-08	NA
	TN	IG100012299	A01	34375581	0/1	0.49	2.21E-09	7.62E-26	3.45E-08	8.18E-08	NA	1.53E-18
		IG100359692	A02	123198708	0/1	0.16	1.05E-03	NA	1.32E-16	NA	7.24E-08	3.45E-10
		IG100278405	A06	59780114	0/1	0.13	1.33E-07	NA	6.75E-14	NA	3.71E-23	NA

*Markers crossed the threshold levels of both the BLINK (P < 1.00E-05, combined) and Multiple Loci Mixed linear Model MLMM (P < 0.01) are reported. AC, assembled chromosome; Pos., position in a chromosome; WS-RF, wet season under rainfed condition; DS-MWS, dry season under moderate water stress condition; DS-SWS, dry season under severe water stress condition.*

#### Wet Season-Rainfed

A total of 11 markers associated with TFW, PH, LL, LW, TN, ST, and IL were identified under the WS-RF condition (Table 3 and Supplementary Figure 8A). The 11 markers were distributed across the seven ACs, A01, B01, B02, A05, B03, B05, and B06. Three markers on B02 and B06 were associated with TFW, two SNP markers at the bottom of B02 showed the strongest association (*P* < 1.25E-12). For PH, one marker on B01 showed a strong association (*P* < 8.29E-20). Similarly, one marker (IG100007430) on B03 was associated with both LL and LW traits. The marker was also strongly associated (*P* < 5.38E-30) with ST. Another marker (IG100222553| F| 0-48:C > T-48:C > T) on B02 was associated with ST. For TN, two markers on A01 and A05 showed strong associations (*P* < 2.50E-13). One marker on B05 showed a strong association with IL.

#### Dry Season-Moderate Water Stress

Seven markers showed an association under moderate water stress conditions in the dry season (Table 3 and Supplementary Figure 8B). The seven markers were associated with PH, LW and TN and were distributed across A01, B01, B02, A04, and A07. Three markers on A01 showed the strongest association with LW (*P* < 9.52E-19) and TN (*P* < 5.15E-11).

#### Dry Season-Severe Water Stress

Under severe water stress conditions in the dry season, a total of 17 markers showed an association with TFW, TDW, WUE, PH, LW and TN. The markers were distributed across ACs, except for B01, B04 and B07. Two markers on B02 and B06 showed an association with the two most important agronomic traits, TDW and WUE. The strongest association with WUE was observed for two markers on B02 (*P* < 4.15E-14) and B06 (*P* < 4.44E-16). Another marker on B02 showed the strongest association (*P* < 9.46E-19) with TFW (Table 3 and Supplementary Figure 8C).

A total of eight markers showed an association with the morphological traits (PH, LW, and TN), of which the strongest association was observed for one marker on A07 with PH, one marker on B06 with LW and another three markers on A01, A02, and A06 with TN (Table 3).

#### Consistency of Marker Associations Across the Two Years

The consistency of the associated markers was tested by separate analysis for each of the 2018 and 2019 data. A total of 10 markers associated with TFW (3), LL (1), LW (1), TN (3), TDW (1), WUE (1) were also identified to be associated with the trait in an individual year. One marker (IG100292170| F| 0-44:C > T-44:C > T) on B06 was consistently associated with TFW, TDW and WUE traits in both years. The strongest consistency, however, was observed for the association of TN at the top of A01 (Figure 4 and Table 3). Some more markers that showed consistent associations with traits across the three soil moisture conditions (WS-RF, DS-MWS, and DS-SWS) based on the MLM model in GWASpoly package (Rosyara et al., 2016) are shown in Supplementary Table 3.

### Markers Associated With Quantitative Trait Loci Governing Purple Pigmentation

**FIGURE 5:**
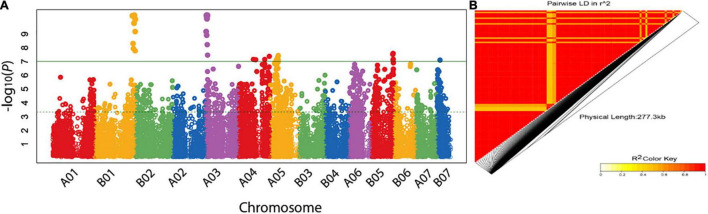
Manhattan plot showing the association of markers with purple color **(A)**. Markers are on the x-axis for each of the 14-assembled chromosomes (AC). The –log10 of *P* values are plotted on the *y*-axis. The threshold level was set at *P-*value> the –log10 value of 7. In **(B)**, the LD heatmap for the pairwise LD of the markers associated with purple color. As indicated by the color key, decreasing and increasing LD is indicated by yellow and red colors, respectively.

A total of 125 markers (23 SNPs + 102 SilicoDArTs) showed an association (Figure 5A, Table 4; and Supplementary Table 1) with the purple-color pigmentation (Supplementary Figure 3) by a marker-trait association analysis using the non-parametric univariate Fisher’s exact test (Warner, 2013). Of the 125 markers, 112 markers (97 SilicoDArTs and 15 SNPs) perfectly discriminated/diagnosed the seven purple genotypes. Of these markers, eight were on B01 and 19 on A03 (Table 4), while 70 were mapped only on contigs (Supplementary Table 1). However, there was no genomic position information for the remaining 15 markers (Supplementary Table 1).

**TABLE 4:** Diagnostic markers for purple color.

Marker	AC	Pos	allele	*P*-value	Status^1^	Protein name
9994171	B01	190390682	C/A	4.39E-11	CA	probable serine/threonine protein kinase
30282538	B01	181775730	C/A	4.71E-11	CA/AA	NA
23641768	B01	185828246	C/G	5.05E-11	CG	glucan endo-1,3-beta-glucosidase 3
23640854	B01	195134132	C/G	8.39E-11	CG/GG	sucrose transport protein
10000565	B01	189376936	G/A	1.62E-10	GA/AA	NA
100013546	B01	194658440	0/1	6.59E-10	present	bifunctional epoxide hydrolase 2-like
100254243	B01	184648641	0/1	4.90E-09	present	NA
100013603	B01	186485183	0/1	1.14E-08	present	cysteine-rich receptor-like protein kinase 6
100205233	A03	2091285	0/1	4.39E-11	present	F-box/kelch-repeat protein
100013579	A03	3641263	0/1	4.39E-11	present	nuclear transcription factor Y subunit A-10
100013690	A03	4065302	0/1	4.39E-11	present	glycine-rich protein DOT1-like
100248653	A03	4447543	0/1	4.39E-11	present	glucan endo-1,3-beta-glucosidase 3
30291207	A03	5016898	G/A	4.39E-11	GA/AA	Remorin family protein
100013697	A03	5042774	0/1	4.39E-11	present	E3 ubiquitin-protein ligase
100013640	A03	5137129	0/1	4.39E-11	present	diphosphomevalonate decarboxylase MVD2, peroxisomal-like
100013671	A03	5189436	0/1	4.39E-11	present	hypothetical protein SEVIR_3G4NA32NANAv2
9897756	A03	6133035	T/C	4.39E-11	TC/CC	NA
23641769	A03	4445594	T/C	4.71E-11	TC/CC	glucan endo-1,3-beta-glucosidase 3
23636057	A03	5305525	A/C	4.71E-11	AC/CC	NA
30279984	A03	3860473	A/G	5.05E-11	AG/GG	NA
100238305	A03	5456675	T/C	5.05E-11	TC	CASP-like protein 1B1
23618539	A03	2845425	C/T	5.42E-11	CT/TT	NA
30291523	A03	4425113	C/T	7.78E-11	CT/TT	Cysteine-rich receptor-like protein kinase
100084872	A03	2094328	0/1	6.59E-10	present	protein NUCLEAR FUSION DEFECTIVE 4
100013725	A03	5362130	0/1	6.59E-10	present	pyridine nucleotide-disulfide oxidoreductase domain-containing protein
23619969	A03	2925440	C/G	1.23E-09	CG/GG	NA
100175270	A03	5573326	T/C	5.87E-09	TC/CC	NA

*Markers genomic position, P-value, marker status, and protein associated with the marker sequences are shown. Additional diagnostic markers, but without genomic position information, are found in Supplementary Table 1. ^1^Status = homozygous/heterozygous in the case of SNPs or present/absent in the case of SilicoDArTs, on purple genotypes; AC, assembled chromosome; Pos, position on AC; NA, protein name information is not available.*

All the 97 SilicoDArT markers were present in the genotypes with purple color and absent in the green genotypes. Three out of the 15 SNP markers were in a heterozygous form (for the allele associated with purple color) in all the seven purple genotypes and were in a homozygous form (for the alternative allele) in the green genotypes. Twelve SNP markers were also in the heterozygous form (for the allele associated with purple color) in six of the purple genotypes, however, they were in the homozygous form in one purple genotype (CNPGL_92-133-3). The markers were in very strong LD representing large haplotype blocks on B01 and A03 (Figure 5B), which were the two ACs with high synteny potentially representing the B and A′ genomes of Napier grass (Yan et al., 2021).

### Markers Associated With Quantitative Trait Loci Governing Feed Quality Traits

A total of 39 associated markers, one to eight markers per trait, were detected for each of the eight feed quality traits under the three-soil moisture conditions (Table 5, Figure 5; and Supplementary Figure 9). However, no associated marker was detected for CP in any of the three-soil moisture conditions.

**TABLE 5:** Markers significantly associated with the feed quality traits, their genomic positions, contrasting alleles, and minor allele frequency (MAF).

Soil moisture condition	Trait	Marker	AC	Pos	allele	MAF	BLINK-model *P-*value			MLMM-model *P*-value		
							2018	2019	Combined	2018	2019	Combined
WS-RF	ADF	IG100036297	A02	144684441	0/1	0.42	0.00029	NA	5.74E-19	NA	7.87E-10	1.44E-06
		IG100007552	A07	95734609	0/1	0,47	NA	NA	6.26E-13	NA	NA	1.17E-15
	ADL	IG100310013| F| 0-11:G > A-11:G > A	B02	169022485	G/A	0.28	NA	2.68E-12	1.70E-14	NA	NA	3.17E-22
		M48572_D23546094	A04	122999396	0/1	0.17	2.07E-13	NA	NA	1.71E-16	NA	NA
	ash	D23592459	A01	157193247	0/1	0.10	4.30E-05	NA	5.19E-09	4.06E-10	NA	1.59E-08
		IG100039072	B02	2111554	0/1	0.34	NA	1.11E-09	2.71E-10	NA	4.56E-07	NA
		IG100033583	A03	6483230	0/1	0,46	1.89E-05	NA	5.58E-08	NA	8.03E-05	NA
	DM	IG100294731| F| 0-41:G > T-41:G > T	A01	189704019	G/T	0.42	1.51E-09	NA	1.68E-12	2.28E-10	NA	NA
		IG100107307| F| 0-32:C > T-32:C > T	B05	89635856	C/T	0.16	1.02E-08	NA	3.47E-11	3.36E-09	NA	NA
	IVOMD	IG100036297	A02	144684441	0/1	0.42	NA	NA	1.24E-19	NA	1.91E-05	NA
		D30280676| F| 0-37:G > A-37:G > A	A04	77484986	G/A	0.20	NA	NA	2.47E-07	5.32E-05	NA	4.65E-05
		IG100097764| F| 0-22:A > T-22:A > T	B03	112819745	A/T	0.12	NA	NA	3.09E-09	NA	6.14E-05	6.12E-12
	NDF	D23601192	B03	27798065	0/1	0.20	NA	0.0026	2.33E-08	NA	6.06E-07	1.78E-16
		IG100092231	B04	17161059	0/1	0.26	NA	4.63E-08	4.20E-07	2.72E-05	NA	NA
		IG100315926| F| 0-19:A > C-19:A > C	B05	19403458	0/1	0.21	NA	2.93E-08	1.21E-07	NA	NA	3.00E-07
	OM	D23572727	B01	160393045	0/1	0.16	NA	0.00014	5.06E-11	NA	2.25E-04	NA
		D23634207| F| 0-40:T > G-40:T > G	B02	11864366	T/G	0.41	9.23E-08	NA	9.75E-09	NA	2.96E-16	1.56E-08

DS-MWS	ADL	D23637168	A04	15533217	0/1	0.45	1.34E-08	0.0061	4.51E-10	5.76E-09	NA	1.12E-08
		IG100219228	B04	88853282	0/1	0.12	NA	0.00025	4.38E-08	NA	NA	3.87E-05
		IG100118601	B03	121019509	0/1	0.38	4.08E-08	3.63E-08	1.14E-08	7.70E-10	8.43E-10	1.15E-18
	ash	IG100261206	B04	9826505	0/1	0.23	NA	4.62E-05	2.89E-07	NA	8.80E-17	5.91E-10
		IG100356234	B07	393133	0/1	0.06	NA	0.0038	1.11E-12	NA	3.01E-10	1.87E-05
	DM	IG100186011	A01	189725890	0/1	0.37	1.18E-09	5.05E-09	7.59E-11	3.25E-05	NA	2.31E-30
	Me	D23602964	A01	168319043	0/1	0.36	0.0042	3.94E-07	5.92E-11	NA	2.47E-07	NA
	NDF	IG100261206	B04	9826505	0/1	0.23	4.89E-08	3.54E-12	3.41E-17	NA	3.31E-19	3.16E-15
	OM	IG100261206	B04	9826505	0/1	0.23	0.00071	0.00011	2.87E-07	NA	8.80E-17	7.02E-13
		IG100356234	B07	393133	0/1	0.06	0.0049	NA	1.09E-12	NA	9.42E-15	NA

DS-SWS	ADF	IG100200286| F| 0-55:G > A-55:G > A	A02	142268235	G/A	0.34	0.0095	NA	1.86E-08	NA	NA	1.81E-08
		IG100189631	B04	93330229	0/1	0.27	6.14E-05	6.68E-05	4.39E-10	NA	NA	1.65E-12
	ADL	IG100267383| F| 0-46:T > C-46:T > C	A04	32856195	T/C	0.28	1.94E-09	0.0022	7.09E-14	3.04E-14	NA	1.88E-12
	ash	IG100261206	B04	9826505	0/1	0.23	0.00022	NA	1.27E-13	NA	NA	4.78E-05
		D23549966	B05	13742398	0/1	0.30	NA	8.92E-07	1.13E-09	NA	NA	2.37E-15
	DM	IG100186011	A01	189725890	0/1	0.43	1.59E-11	1.14E-12	1.84E-23	1.59E-07	6.70E-18	1.42E-17
		IG100107707| F| 0-30:A > G-30:A > G	B02	26851818	A/G	0.07	NA	NA	2.26E-10	NA	NA	5.28E-07
		D23620253	A07	12071566	0/1	0.23	7.28E-07	NA	5.57E-09	NA	3.17E-08	NA
	OM	D23604854	B03	101008981	0/1	0.43	4.78E-07	1.33E-11	1.12E-16	NA	1.27E-4	NA
		IG100037685	B06	78317387	0/1	0.07	NA	NA	4.88E-14	1.47E-06	NA	NA
		D30280949	B07	40268843	0/1	0.07	NA	NA	1.81E-10	1.98E-06	NA	NA
		IG100175157	A01	147194660	0/1	0.05	4.42E-06	NA	NA	3.76E-14	NA	2.15E-10

*Markers crossed the threshold levels of both the BLINK (P < 1.00E-05, combined) and Multiple Loci Mixed linear Model MLMM (P < 0.01) are reported. AC, assembled chromosome; Pos., position in a chromosome; WS-RF, wet season under rainfed condition; DS-MWS, dry season under moderate stress condition; DS-SWS, dry season under severe water stress condition.*

Under the WS-RF condition, 17 markers associated with ADF, ADL, ash, DM, IVOMD, NDF, and OM were detected (Table 5 and Supplementary Figure 9A). The distribution of markers was proportional between the A′ and B genomes. Two markers on chromosome A02 and A07 were strongly associated (*P* < 6.26E-13) with ADF. The marker on chromosome A02 was also strongly associated with IVOMD. ADF and IVOMD were strongly negatively correlated (>−0.90) and hence the allele positively affecting ADF will affect IVOMD negatively and vice versa. Only one marker on chromosome B02 was associated with ADL, while three markers on chromosome A01, B02, and A03 were associated with ash.

A total of ten markers associated with ADL, ash, DM, Me, NDF, and OM were detected under the DS-MWS condition (Table 5 and Supplementary Figure 9B). No association was detected for CP, ADF, and IVOMD. One marker on chromosome B04 was associated with ash, NDF and OM, in which ash was strongly negatively correlated with NDF and OM. Similarly, one marker on chromosome B07 was associated with both ash and OM. Seven out of the ten markers were from the B genome.

Under the DS-SWS condition, twelve markers were associated with ADF, ADL, ash, DM, and OM, but no association was detected for CP, Me, NDF, and IVOMD (Table 5 and Supplementary Figure 9C). Seven out of the twelve markers were from the B genome.

**FIGURE 6:**
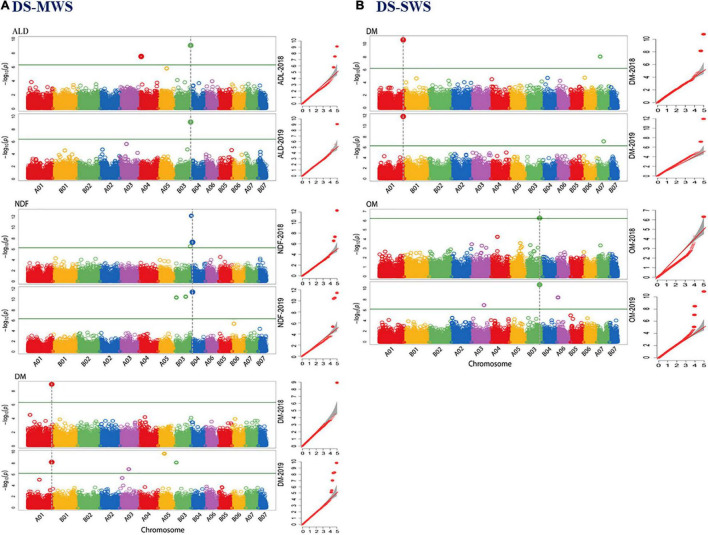
Manhattan and quantile-quantile (Q-Q) plots of markers that showed overlapping associations for 2 years data of the nutritional quality traits in dry season under moderate water stress (DS-MWS) **(A)**, and dry season under severe water stress (DS-SWS) **(B)** conditions. Markers are on the *x*-axis per the 14-assembled chromosomes (AC). The –log10 of *P* values are plotted on the *y*-axis. The traits are shown at the left: acid detergent lignin (ADL), dry matter (DM), neutral detergent fiber (NDF), and organic matter (OM).

In a separate analysis for the individual years data, 10 markers associated with DM (2), OM (2), Me (1), ADF (1), ADL (3), and NDF (1) were consistent, showing an association in both years. Among these, the marker at approximately 190Mbp at the bottom of chromosome A01 and associated with the DM was the most consistent (Table 5). The markers that showed more consistent associations across the 2 years are shown in Figure 6.

### Co-localization and Functional Description of Sequences Associated With the Significant Markers

Overlapping genomic positions of associated markers and co-localization with known QTLs can be used as an additional criterion to determine genomic regions controlling the traits and for the identification of candidate genes. Out of the six markers associated with TFW, three markers that showed strong associations were on B02. Two of these markers were detected under WS-RF and were located at the bottom of B02, while the third marker, located at the top of B02, was detected in the DS-SWS condition. These marker/trait associations indicated that genomic positions from 138 to 176 mbp at the bottom of B02 and at around 7 mbp at the top of B02 might harbor QTLs controlling TFW under wet and dry conditions, respectively. The two markers at the bottom of B02 were associated with genes encoding a scavenger receptor class F member 2-like and a putative box C/D snoRNA protein. The one at the top of B02 was associated with a gene encoding GDSL esterase/lipase protein (Supplementary Table 2).

**FIGURE 7:**
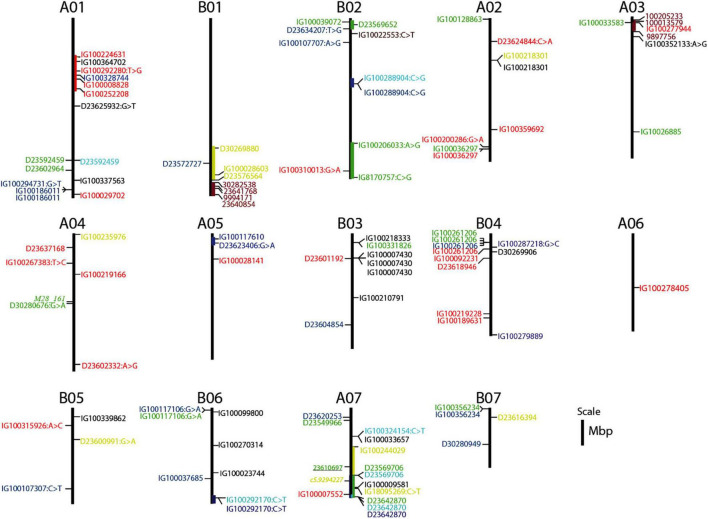
Genomic map position of markers associated with QTLs governing the agronomic, morphological, and feed quality traits. Markers associated with the agro-morphological traits are shown to the right of the assembled chromosome (AC), while markers associated with the feed quality traits are shown to the left of the AC. Markers reported previously for *Setaria italica* (Jaiswal et al., 2019b; italicized) and pearl millet (Varshney et al., 2017; underlined) are also shown to the left of the AC. Markers strongly associated with TFW (total fresh weight), TDW (total dry weight), WUE (water use efficiency), PH (plant height), TN (tiller number), and PP (purple pigmentation) are shaded by green, light-blue, deep-blue, yellow, red, purple colors, respectively. Markers associated with traits that are affecting positively the feed nutritional quality (IVOMD, Me, and ash) are shaded green, while markers associated with traits linked with fiber and lignin components (ADF, ADL, and NDF) are shaded red. The markers associated with OM and DM are shaded blue.

The regions on B02 also contained markers associated with TDW and WUE, which were highly correlated with TFW. Two markers on B02 and B06 showed an association with both TDW and WUE. However, both these traits were strongly associated with markers at the bottom of B06 (at around 100Mbp) and a second stronger association at the top of A05, at about 10 Mbp (Figure 7). A high level of synteny between B02 and A07, and, between A05 and B06 has been reported (Yan et al., 2021). A pearl millet marker associated with total fresh weight (Varshney et al., 2017) was mapped at the bottom of A07 (Figure 7). The marker on B02 was associated with a gene encoding a 60 kDa jasmonate-induced protein which is involved in abiotic stress responses and defense against pathogens, while the marker on A05 was associated with a gene encoding a prolyl 4-hydroxylase protein (Supplementary Table 2).

Out of the 8 markers associated with TN under the three soil moisture conditions, four were on A01. Of these, two were detected by both the BLINK.R and MLMM models across the 2 years and located at the top of A01 within the genomic region between 59 to 67 mbp (Table 3), suggesting the position of a major QTL controlling TN. Two out of the four markers were associated with genes encoding a xylanase inhibitor protein and protein DMP10 (Supplementary Table 2). DMP proteins are plant specific membrane proteins which function function in various physiological processes such as reproductive development and senescence in plants (Zhu et al., 2021).

The analysis of marker-trait associations for the morphological traits was more complex compared to the agronomic traits. For PH, a greater number and stronger association were detected on B01 and A07. Genomic regions spanning approximately 29 mbp at the bottom of B01 and 46 mbp on A07 could be more important for the control of this trait. Four of the markers were associated with genes encoding a 23 kDa jasmonate-induced protein, protein SHORT-ROOT 1, F-box/FBD/LRR-repeat protein and an antifreeze protein (Supplementary Table 2). The marker on A07 was co-localized with a marker previously shown to be associated with PH in *Setaria italica* (Jaiswal et al., 2019b). This region also harbors two markers associated with LL. These two markers were associated with genes encoding an ABC transporter B family member and a disease resistance protein kinase. However, the strongest association with LL was with a marker at the top of B03 at around 27 Mbp, which was also associated with LW and ST (Figure 7).

Genomic-position information was found for only 27 of the markers associated with the purple genotypes and all of them were mapped at the bottom of B01 and the top of A03, which are ACs with high synteny (Yan et al., 2021). This indicates that genomic regions spanning about 13 mbp at the bottom of B01 and about 5 mbp at the top of A03 harbor QTLs which govern the purple pigmentation in Napier grass. The two markers on B01 were co-localized with the markers associated with PH and another five markers on A03 were co-localized with TN and LL (Figure 7). Thirty of the markers were associated with genes encoding functional proteins (Table 4 and Supplementary Table 1). One gene was annotated as an anthocyanin regulatory R-S protein and the annotation of most other genes was related to plant disease resistance.

A marker associated with ash at the top of B02 and another marker associated with ADL at the bottom of B02 were co-localized with QTL regions controlling TFW under dry and wet conditions, respectively. Another marker associated with ash at the top of A03 was co-localized with the QTL region governing the purple pigmentation (Figure 7). The genomic region spanning about 22 kbp at the bottom of A01 harbors QTLs controlling DM in all the three soil-moisture conditions. One of the markers was associated with a gene encoding a MADS-box transcription factor, which is a member of regulatory networks and governs diverse developmental processes in plants, including root development (Liu et al., 2020). A marker associated with IVOMD on A04 was co-localized with M28_161, which is a marker associated with high values of biomass digestibility in a previous study (Rocha et al., 2019).

## Discussion

Genome-wide association study (GWAS) scans genetic variation across the whole genome to find signals of associations with a variation in phenotypic expression for various complex traits and is an efficient approach as it does not need the development of specific crosses, which is time consuming (Flint-Garcia et al., 2003; Huang and Han, 2014). Instead, it uses existing collections of genotypes that enables targeting a broader and more relevant genetic spectrum for plant breeders (Huang and Han, 2014). Hence, by linking markers to traits and traits to genotypes, GWAS is well suited for the genetic characterization and exploitation of genebank collections. However, for successful application of GWAS, an accurate phenotyping of the population and, as much as possible, estimating the accurate genetic value of individual genotypes is mandatory. In addition, high-density genome-wide markers and an appropriate model selection to avoid false positives are also key requirements.

### Prior and Post Phenotype Data Corrections for Spatial Variations

Obtaining accurate estimates of the genetic value of an individual genotype is central to the identification of markers associated with QTLs. Within an experimental field, many factors combine to generate microenvironments that vary from plot to plot and across blocks (Rodríguez-Álvarez et al., 2018), affecting biomass yield and other morphological and feed quality traits. Hence, it is important to correct for those factors when estimating genotypic effects. In this study, *a priori* and *a posteriori* spatial variability control measures were employed. As an *a priori* control measure, we used a subset of a diverse set of Napier grass genotypes, a p-rep experimental design (Williams et al., 2011) in four replications, randomization in each block, as well as check genotypes that were duplicated per block. In addition, border row plants surrounding each block were used to reduce the border effects and trait measurements were averaged from six harvests over a 2-year period. However, it has been reported that these conventional control measures are not enough to account for the fine-grained spatial variability within blocks, in particular, the conventional control measures do not account for dependency between neighboring blocks and plots within blocks, which can affect the estimation of genetic values (Lado et al., 2013; Velazco et al., 2017; Elias et al., 2018; Rodríguez-Álvarez et al., 2018). Hence, as a post data correction measure, we used the open-source model of Rodríguez-Álvarez et al. (2018) that uses two-dimensional smooth surfaces along the rows and columns of the experimental blocks to capture spatial variations. The model also allowed us to correct the phenotypes for heterogeneity in soil moisture content, soil nutrient parameters, and multi-harvests, which were used as fixed covariates in the spatial analysis. The model was able to show variation in phenotypic data due to spatial variation within a block as well as across blocks and accordingly allowed us to adjust the phenotype data, which improved the quality of our data and increased the precision in the estimation of trait heritability. A substantial improvement in heritability estimation was observed for IL, IVOMD, CP, and Me, with up to 114, 109, 90, and 68% improvement recorded, respectively. Similar trends of an increase in heritability estimate of traits through the application of a spatial analysis have been reported in wheat (Lado et al., 2013), sorghum (Velazco et al., 2017), and cassava (Elias et al., 2018). A greater heritability implies that a greater proportion of the phenotypic variance in the experiment was due to genetic differences among genotypes (Visscher et al., 2008), which is important for QTL identification by GWAS and other genetic analysis approaches.

### High-Density Genome-Wide Markers Mapped Onto the Napier Grass Genome

Another important prerequisite of GWAS is the availability of high-density genome-wide distributed markers, which has been a challenge especially in orphan crops which include many tropical forages. The genotyping by sequencing (GBS) method (Jaccoud, 2001; Kilian et al., 2012) applied in this study provided high density genome-wide dominant (silicoDArT) and co-dominant (SNP) markers and was found to be reliable, efficient and cost effective. The detail of the marker’s polymorphism and other related information was reported previously (Muktar et al., 2019). However, until recently we have been using the pearl millet (*Cenchrus americanus*) genome (Varshney et al., 2017) to identify the genomic positions and genome-wide distribution of the markers. Subsequently, only 17% of the silicoDArT markers and 33 to 39% of the SNP markers were able to be mapped on the seven chromosomes of pearl millet (Paudel et al., 2018; Muktar et al., 2019). Fortunately, a Napier grass reference genome was made public recently (Yan et al., 2021) which enabled us to generate much more information, including genomic position and genome-wide distribution for most of the markers and an enhanced estimation of LD and LD-decay in the Napier grass genome. More than 90% of the SNP and 73% of the silicoDArT markers were able to be mapped onto the fourteen assembled chromosomes (AC) of the Napier grass genome. The markers were evenly distributed across the genome with some gaps around the middle part of each AC, which probably represents the gene-poor regions of centromeres (Bennetzen et al., 2012; Varshney et al., 2017). However, the density and distribution of the markers was by no means comprehensive, and this resource does not represent the extensive genome coverage required to detect all the possible QTLs in the Napier grass genome. Rather it represents about a quarter of the estimated marker-density and QTL detection power in Napier grass, as estimated using the average LD-decay across the genome previously (Muktar et al., 2019) and in the present study. In this study, similar to the previous report (Muktar et al., 2019), the LD decayed very rapidly, on average at about 3.48 kbp, and varied across the ACs. A long LD block was observed in A01, while the shortest one was in B07. Interestingly, the number of markers in each AC was proportional or positively correlated with the average LD-decay distance per AC. Detailed information about LD and LD-decay across the Napier grass genome and LD variation between collections can be found in Muktar et al. (2019).

### Association Analysis and Correction for Population Structure and Cryptic Relatedness

The third important concern in GWAS is the presence of population structure and cryptic relatedness in the mapping panel that could prevent the association analysis from correctly identifying the true marker-trait association and could lead to the identification of false-positive or spurious associations (Flint-Garcia et al., 2003; Huang and Han, 2014). Therefore, it is important to include population structure and pairwise kinship matrix as covariates and to select the appropriate statistical models that can sufficiently deal with those factors. The detail of population structure and genetic diversity in our Napier grass collections was reported previously (Muktar et al., 2019), in which five to seven subpopulations were detected using different genetic diversity study approaches. A similar population stratification with three to six clusters was seen in the 84 genotypes used in this study (Supplementary Figure 10), in which the pairwise Nei’s genetic distance ranged from 0.02 to 0.81 with an average value of 0.44.

Population size is another important factor in GWAS, and a larger sample size increases the power of detecting QTLs and the map resolution, particularly for the quantitative traits (Korte and Farlow, 2013). However, in this study, we used a total of 84 genetically diverse genotypes, focusing on genebank material from the ILRI and EMBRAPA collections that have no restriction on their future use in order to ensure maximum accessibility and uptake of our outputs. Consequently, 84 was the maximum number of genotypes that were available to us for field phenotyping. We systematically selected the 84 accessions based on their genetic distance and tried to ensure their representativeness of the genetic diversity. Although it is considered that the larger the sample size, the better result, it has been shown that some meaningful results can be obtained with less than 100 accessions and QTLs can be detected that are controlled by a few loci that explain a larger portion of the phenotypic variance (Korte and Farlow, 2013).

For the morphological, agronomic, and feed quality quantitative traits, we employed two different GWAS models implemented in the Genomic Association and Prediction Integrated Tool version 3 (GAPIT3) (Wang and Zhang, 2021). The BLINK.R (Bayesian-information and Linkage-disequilibrium Iteratively Nested Keyway) (Huang et al., 2018) model is based on linkage disequilibrium (LD) and controls confounding issues arising due to cryptic relatedness and multiple testing corrections by using all tested markers within an LD block. Furthermore, the model takes population stratification information as covariates and hence the first three to five PCs from a PCA analysis were used in our analysis. The model efficiency in controlling the above-described confounding factors was demonstrated by the quantile-quantile (Q-Q) plots, which showed a similar distribution of observed and expected *p*-values along the diagonal line for most of the markers and a sharp curve at the end of the line representing a few associated markers. The multiple-locus mixed linear model (MLMM) (Segura et al., 2012) takes into account both population structure and pairwise kinship matrix to avoid false positives. The two models were complementary to each other in that both identified many common markers and a few different markers associated with the traits. There were a few QTL regions detected only by the BLINK.R but not by the MLMM, and vice versa, which could be attributed to the differences in algorithms and parameters used in the two models. However, we reported here markers detected by both models to optimize the selection of QTL regions and candidate genes for further exploration. Hence, more than 35 and 39 independent markers associated with the agro-morphological and nutritional quality traits, respectively, under the three different soil moisture conditions were identified by both models.

### Markers Associated With Forage Biomass and Water-Use Efficiency Traits

Genome-wide association study identified six QTL regions associated with TFW, which represents the above-ground fresh biomass production. The QTLs at the bottom (about 138 mbp) of B02, top of B03 (11 mbp), and bottom of B06 (100 mbp) could be more interesting for breeding applications as they showed a strong and consistent association in each year and were detected by both the BLINK.R and the MLMM models. The QTLs at the top of B02 were associated with increased biomass yield under severe water stress conditions in the dry season, while those at the bottom were associated with high biomass production during the wet season. The QTLs at the bottom of B02 were linked with genes encoding scavenger receptor and box C/D snoRNA proteins, which are proteins involved in immune response and rRNA modification (Streit et al., 2020). In our previous marker-trait analysis (Habte et al., 2020), we reported markers associated with annual dry weight yield at the bottom of A03 (14 markers), B05 (14 markers), and B02 (4 markers).

Three QTL regions around the middle of A05 (about 80 mbp), B02 (73 mbp) and bottom of B06 (100 mbp), showed an association with TDW and, specifically, increased total dry matter production under the SWS conditions in the dry season. These three QTLs were also associated with improved WUE, which is the most important factor in improving productivity under limited water availability in the dry season and in addressing the current challenges to forage production due to climate change. The QTL at the bottom of B06 could be more interesting as it was consistently identified across both years. High synteny and co-linearity between B06 and A05 chromosomes has been reported (Yan et al., 2021). Hence, we can speculate that these two ACs may represent the A′ and B homeologous chromosomes of Napier grass. Napier grass is an allotetraploid grass (2n = 4x = 28) with a complex genome (A′A′BB), with the A′ genome showing a high degree of homology with the pearl millet (2n = 2x = 14 with AA genomes) A genome (Dos Reis et al., 2014). The QTL on B02 was linked with a gene encoding a 60 kDa jasmonate-induced protein which is involved in abiotic stress responses and defense against pathogens (Rustgi et al., 2014), while the marker on A05 was linked with a gene encoding a prolyl 4-hydroxylase protein, which is involved in plant growth and development and in responses to abiotic stresses (Vlad et al., 2007). The marker on A05 was mapped on pearl millet linkage group 2, and co-localized with the major drought-tolerant QTL (DT-QTL) reported in pearl millet (Tharanya et al., 2018).

A QTL at the bottom of B06 was consistently and strongly associated with the three most highly correlated traits (TFW, TDW, and WUE) indicating the tight linkage between the agronomic and water use efficiency traits. Co-mapping and tight linkage of QTLs for agronomic and water use efficiency traits were reported in pearl millet, in which close linkages between QTLs controlling the traits in four genetic regions of linkage group 2 were detected (Tharanya et al., 2018). The possibility of a pleiotropic effect, in which the three traits are controlled by a single QTL, was reported in *Setaria italica* (Feldman et al., 2018). Co-localization of QTLs and the marker associated with them can potentially be exploited in marker-assisted selection to develop high biomass producing Napier grass varieties for dry and water deficit areas.

### Markers Associated With Morphological Traits

In this study, a greater number of QTL regions associated with the morphological traits (PH, LL, LW, ST, IL, and TN) were detected by a total of 23 markers, which might suggest that the genetic architecture is more complex when compared to the architecture of the agronomic traits. Among these, the QTL region at the top of A01 (at around 60 mbp) was strongly and consistently associated with TN under the three soil moisture conditions in both years, and hence is considered a major QTL. Higher tillering ability is an important trait in establishment and regrowth in grasses (Pereira et al., 2013) and was positively correlated with the TFW, TDW, and WUE traits while showing no correlation with traits linked with fiber and lignin (ADF, NDF and ADL), indicating its importance in the high biomass production of Napier grass without compromising the feed nutritional quality.

We also identified two QTL regions for PH at the bottom of B01 and A07, one QTL region at the top of B03 controlling LL, LW, and ST, and another QTL region for IL at the top of B05. The markers associated with the QTLs will be useful in a Napier grass improvement program through the application of marker-assisted selection. The QTL region for PH on B01 is co-localized with the QTL region associated with purple pigmentation. A similar result was reported in pearl millet (Azhaguvel et al., 2003), in which loci controlling purple foliage color, dwarf plant height, and resistance to downy mildew and rust diseases were co-localized on pearl millet linkage group 4. The QTL region associated with PH on A07 was co-localized with a marker associated with PH in *Setaria italica* chromosome 5 (Jaiswal et al., 2019b), which shows a high degree of synteny and co-linearity with the top part of the Napier grass A07 (Yan et al., 2021).

By using sequence tags corresponding to the associated markers, we identified several candidate genes linked to the QTL regions. Two markers in the QTL region associated with TN were linked with genes encoding a xylanase inhibitor protein and a protein DMP10. In the QTL region associated with PH, four markers were linked with genes encoding a 23 kDa jasmonate-induced protein, protein SHORT-ROOT 1, F-box/FBD/LRR-repeat protein and an antifreeze protein. These proteins are involved in abiotic stress responses and in different developmental processes, for example, the SHORT-ROOT protein has been reported to play a role in regulation of primary, lateral, and adventitious root developments in Arabidopsis (Lucas et al., 2011; Rustgi et al., 2014; An et al., 2019). The candidate genes will be useful for further characterization of the QTL regions and potentially cloning of the QTLs using the candidate gene association mapping approach.

### Markers Associated With Purple Pigmentation

For the qualitative purple pigmentation trait, QTL regions were identified by the non-parametric univariate Fisher’s exact test (Warner, 2013) that detected a greater number of significantly associated markers around two genomic regions. Two QTL regions toward the bottom of B01 (around 190 mbp) and the top of A03 (around 2 to 3 mbp) were associated with purple pigmentation in Napier grass. The two genomic regions show a high synteny block (Yan et al., 2021) representing the B and A′ homeologous chromosomes of Napier grass (Dos Reis et al., 2014). Out of the different expanded genes involved in anthocyanin biosynthesis in Napier grass, phenylalanine ammonia lyase (PAL) and chalcone synthase (CHS) were detected on B01 (Yan et al., 2021). One of the markers associated with the QTLs was linked with a gene annotated as an anthocyanin regulatory protein and the annotation of most other genes within the QTL regions was related to plant disease resistance, suggesting the co-localization of the two traits. Anthocyanins are naturally occurring pigments belonging to the group of flavonoids, a subclass of the polyphenol family (Martín et al., 2017), and are involved in biotic and abiotic stress tolerance (Naing et al., 2018). In addition, anthocyanin expression is associated with the expression of stress response genes in plants, such as genes involved in drought, water logging, cold tolerance and disease resistance (Ren et al., 2019).

Our findings are in line with previous reports on pearl millet (Azhaguvel et al., 2003; Varalakshmi et al., 2012), in which they mapped the *P* foliage color locus to pearl millet linkage group 4. The pearl millet linkage group 4 shows strong synteny and co-linearity with the Napier grass A03 and also some synteny with B01 (Yan et al., 2021). In addition, downy mildew and rust resistance genes have been identified in this genomic region of the pearl millet genome (Azhaguvel et al., 2003; Varalakshmi et al., 2012). According to Varalakshmi et al. (2012), the pearl millet purple pigmentation of the leaf sheath, midrib and leaf margin are co-inherited under the control of a single dominant locus (the “midrib complex”) and are inseparably associated with the locus governing the purple coloration of the internode.

The markers identified in this study can be used as tagging markers for map-based cloning of the QTLs controlling purple coloration (anthocyanin) and stress tolerance related genes, which will be very important for the genetic improvement of Napier grass. The markers might also be useful in marker-assisted selection for disease resistance and stress tolerance in Napier grass.

### Markers Associated With Feed Quality Traits

A total of eleven markers, representing different QTL regions across the genome, were identified for IVOMD, Me and ash under the three-soil moisture conditions. These traits were strongly correlated and positively affect the feed nutritional quality. No associated marker was identified for CP, however, this trait was strongly correlated with IVOMD and Me, and hence the markers identified for these two traits could be used in marker-assisted selection for CP. Marker M28_161 that was associated with high values of biomass digestibility in a previous study (Rocha et al., 2019) was co-localized with the marker (D30280676| F| 0-37:G > A-37:G > A) associated with IVOMD on A04, in this study. A genomic region approximately 190Mbp at the bottom of chromosome A01 was consistently and strongly associated with DM, indicating that the region harbors a major QTL controlling the trait. Dry matter is the non-moisture portion of a feed ingredient or diet and contains the essential nutrients within a given forage. Similarly, fourteen markers were detected for ADF, NDF and ADL, which are mainly linked with the cell wall components of fiber and lignin that negatively affect the feed nutritional quality. Genomic regions at the bottom of B03 (approximately 121 mbp) and top of B04 (9 mbp) showed consistent association with ADL and NDF and could be interesting for further exploitation and in improving feed quality in Napier grass through MAS. Improving the digestibility of forages by reducing lignin and fiber is a major goal in forage crop breeding programs (Rocha et al., 2019; Habte et al., 2020).

## Conclusion

1. In this study, we determined the true genetic response of Napier grass genotypes to the morphological, agronomic and feed quality traits by using different approaches that reduced the environmental effects and errors as much as possible. We also showed that the quality of the phenotype data and precision in the estimation of trait heritability can be improved using spatial analysis.
2. Most of the high-density genome-wide markers generated in this study were mapped onto the recently assembled Napier grass genome. Hence, genomic position information was generated for more than 90% of the SNP and 73% of the silicoDArT markers, which improved the identification of genomic regions that harbor QTLs for the important forage traits. The availability of the genome sequence is an important asset and provides a lot of opportunities for the characterization and cloning of the QTLs and for the development of improved Napier grass varieties.
3. Our study led to the identification of at least 35 novel QTL regions and associated markers for morphological, agronomic and water use efficiency traits, and more than 39 novel QTL regions for feed quality traits, and, provided clearer insights into the genetic architecture of the traits in Napier grass. The desirable alleles of the associated markers identified in this study will be useful in Napier grass improvement through marker-assisted breeding. In addition, candidate genes linked with the associated markers were identified and can be used for further characterization and validation of the QTLs through candidate gene association mapping. Further validation of the associated markers, QTLs and the candidate genes identified here may lead to a better understanding of the genetic/genomic bases for the trait’s genetic variation.
4. We also identified two major QTL regions associated with the Napier grass purple color, which is associated with anthocyanin pigmentation. The markers identified in this study can be used as tags for map-based cloning of the QTLs controlling purple pigmentation and stress tolerance related genes, which will be very important for the genetic improvement of Napier grass. Anthocyanin expression is associated with the expression of stress-response genes, such as genes involved in drought, cold and waterlogging tolerance, and disease resistance.
5. The information gained from the present study will be useful for the genetic improvement of Napier grass production with enhanced water use efficiency while maintaining its nutritional quality.

## Data Availability Statement

The genome-wide markers (and the corresponding FASTq files) datasets used in this study were deposited in the ILRI HPC and can be accessed here: https://hpc.ilri.cgiar.org/~mshehabu/napier-grass-GBS-data-2019/. Additional data are available from the corresponding author on reasonable request.

## Author Contributions

CJ designed and supervised the project and the manuscript writing. MM analyzed the data and wrote the manuscript. EH collected the phenotype data. AT and AN collected leaf samples. AT extracted DNA. YA involved in phenotype data collection. K-WL involved in the supervision of the phenotyping project. JZ involved in the supervision of the genotyping project. All authors made a significant contribution to the development of this manuscript and approved it for publication.

## Conflict of Interest

The authors declare that the research was conducted in the absence of any commercial or financial relationships that could be construed as a potential conflict of interest.

## Publisher’s Note

All claims expressed in this article are solely those of the authors and do not necessarily represent those of their affiliated organizations, or those of the publisher, the editors and the reviewers. Any product that may be evaluated in this article, or claim that may be made by its manufacturer, is not guaranteed or endorsed by the publisher.

## Footnotes

1
http://www.diversityarrays.com/

2
https://blast.ncbi.nlm.nih.gov/Blast.cgi

3
https://www.r-project.org/
