# Supplementary material for: Insights Into the Genetic Architecture of Complex Traits in Napier Grass (Cenchrus purpureus) and QTL Regions Governing Forage Biomass Yield, Water Use Efficiency and Feed Quality Traits
Source: Front Plant Sci. 2022 Jan 7;12:678862. doi: 10.3389/fpls.2021.678862 (PMC8776657; doi:10.3389/fpls.2021.678862)
Supplement: Supplementary file 1 [file Data_Sheet_1.docx]

Supplementary Figures

**Supplementary Figure 1.** Volumetric soil water content (VWC) showing the average soil water content (measured using the Delta soil moisture probe (HD), England) per each of the six harvests during wet season under rainfed (WS-RF, harvests 4, 5, 6, 10, 11, and 12), dry-season (DS, harvests 7, 8, 9, 13, 14, and 15) under moderate water stress (DS-MWS), and dry-season under severe water stress (DS-SWS) conditions.


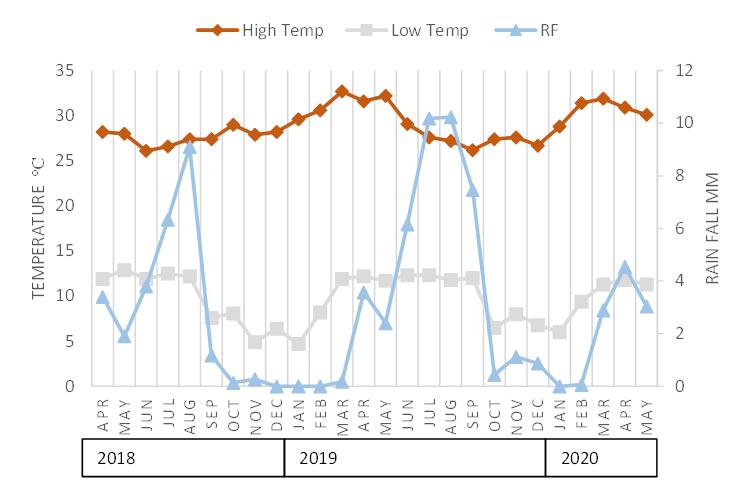


**Supplementary Figure 2.** High temperature, low temperature, and rainfall (RF) data for 2018-2020 at Bishoftu trial site.

B

A


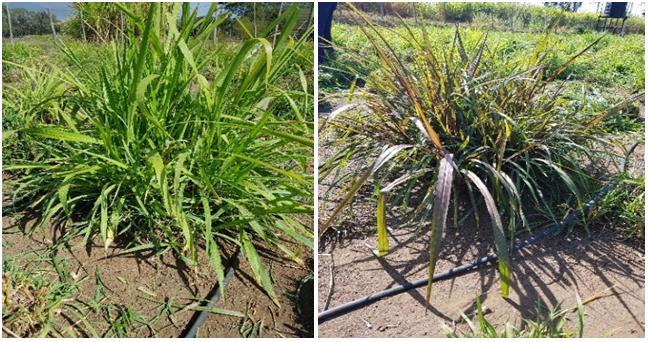


**Supplementary Figure 3.** Green (A) and purple (B) phenotypes of Napier grass genotypes. The purple color is found on leaves, mid ribs, petioles and stems.


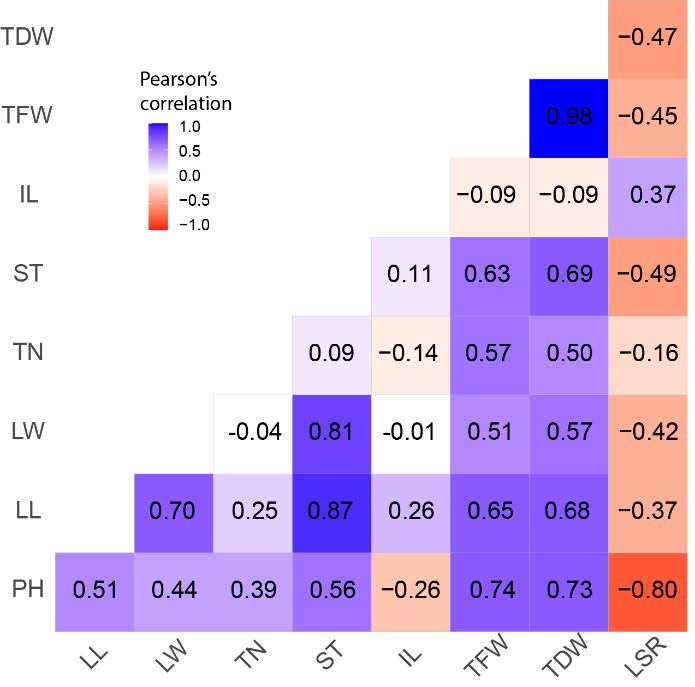

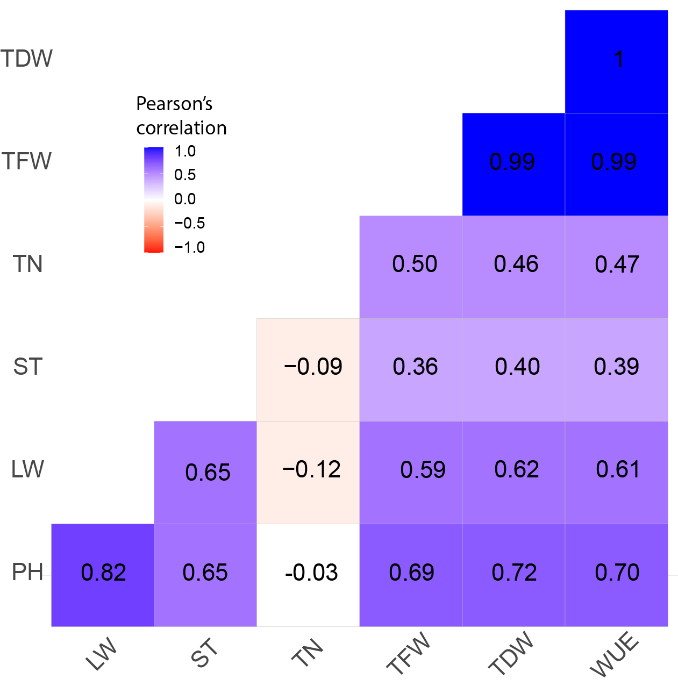

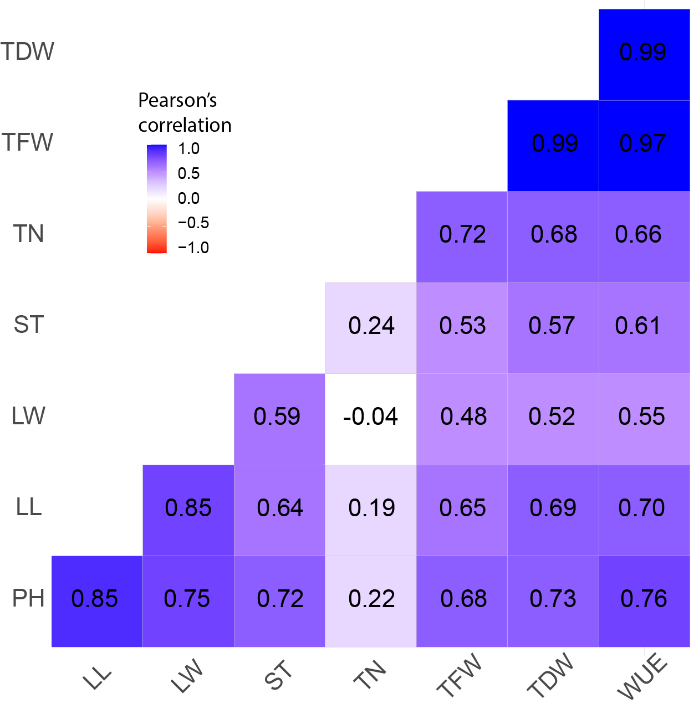


A

B

C

**Supplementary Figure 4.** Correlations among agro-morphological traits. (A) Wet season under rainfed (WS-RF), which is the average of six harvests (harvest 4,5,6,10,11,12) from four blocks; (B) Dry season under moderate water stress (DS-MWS), which is the average of six harvests (harvest 7,8,9, 13, 14, 15) from blocks 1 and 3 (C) Dry season under severe water stress (DS-SWS), which is the average of six harvests (harvest 7,8,9, 13, 14, 15) from blocks 2 and 4.

A

B

C


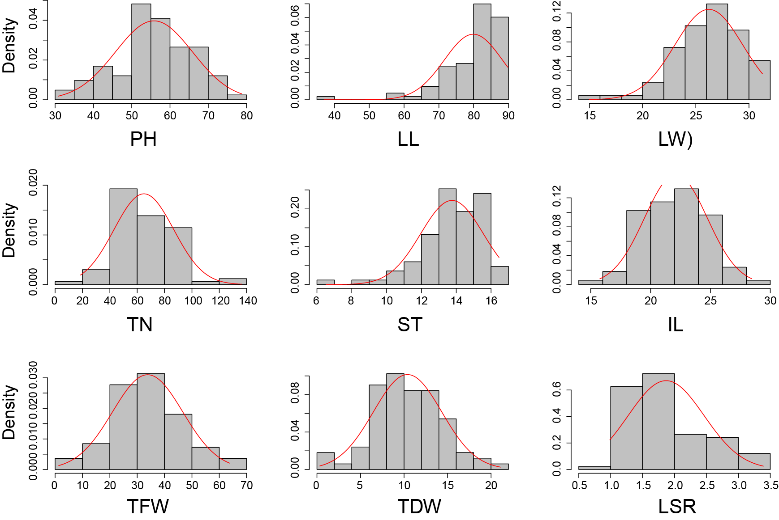

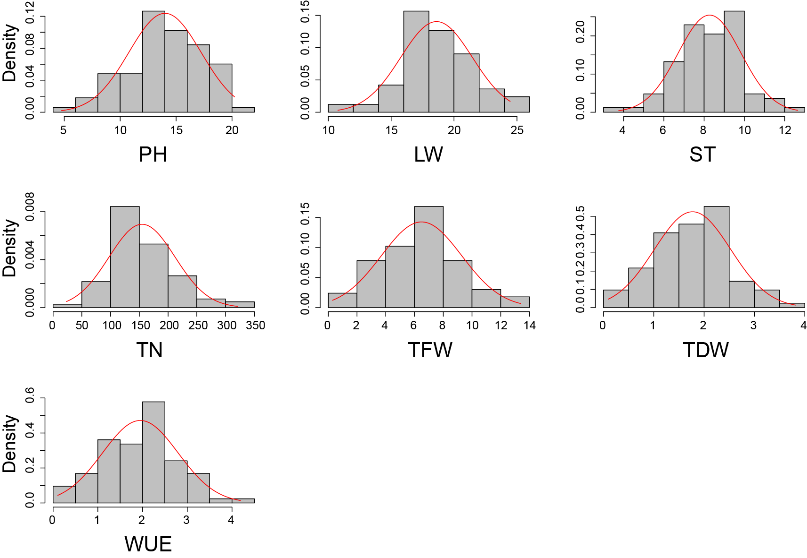

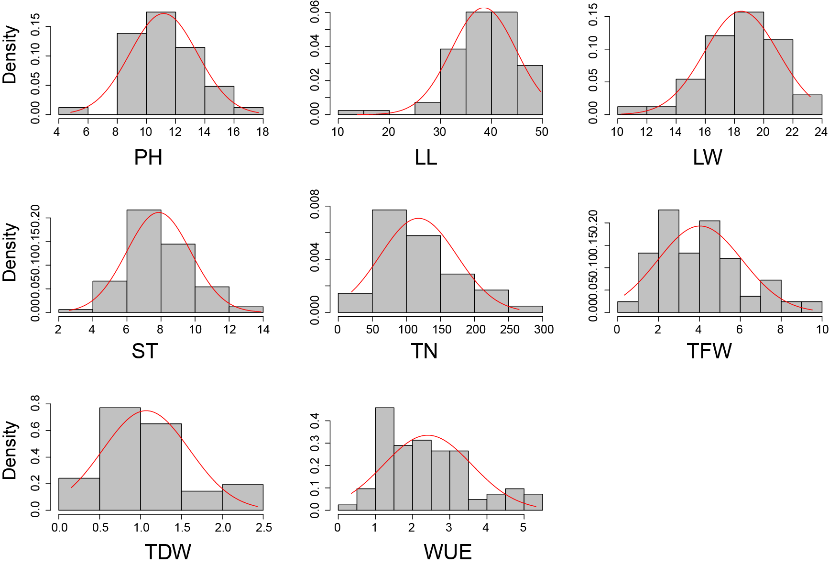


**Supplementary Figure 5**. Distribution of agro-morphological traits (A) Wet season under rainfed (WS-RF), (B) Dry season under moderate water stress (DS-MWS) (C) Dry season under severe water stress (DS-SWS) conditions.

**A**

**B**

**C**

**Supplementary Figure 6.** Correlations among feed quality traits. (A) Wet season under rainfed (WS-RF), which is the average of six harvests (harvest 4,5,6,10,11,12) from four blocks; (B) Dry season under moderate water stress (DS-MWS), which is the average of six harvests (harvest 7,8,9, 13, 14, 15) from blocks 1 and 3 (C) Dry season under severe water stress (DS-SWS), which is the average of six harvests (harvest 7,8,9, 13, 14, 15) from blocks 2 and 4.


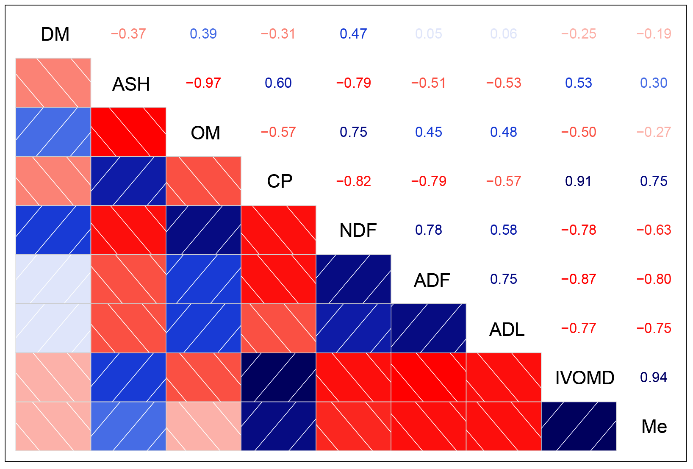

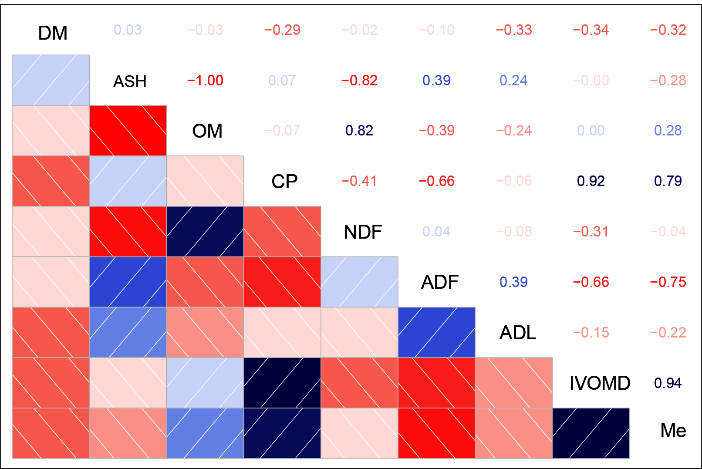

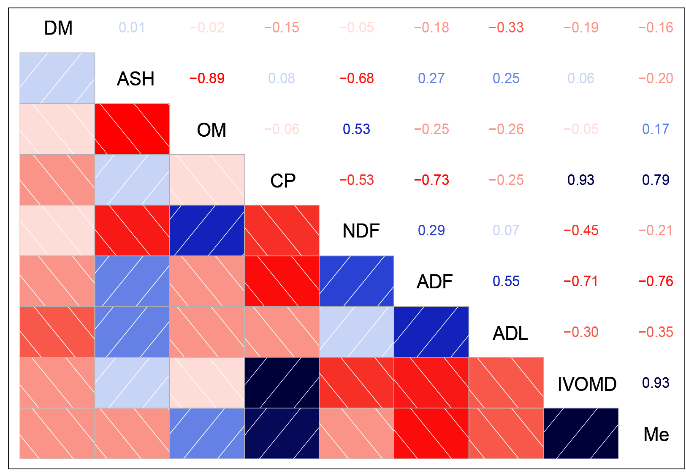


.

**Supplementary Figure 7.** Feed quality traits distribution. (A) Wet season under rainfed (WS-RF), (B) Dry season under moderate water stress (DS-MWS) (C) Dry season under severe water stress (DS-SWS) conditions.

**A**

**B**

**C**


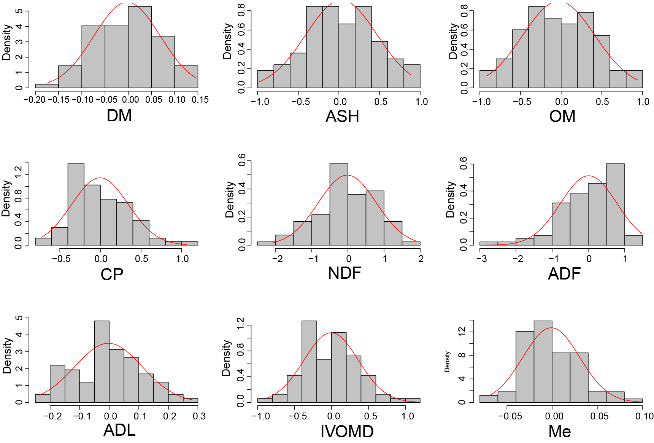

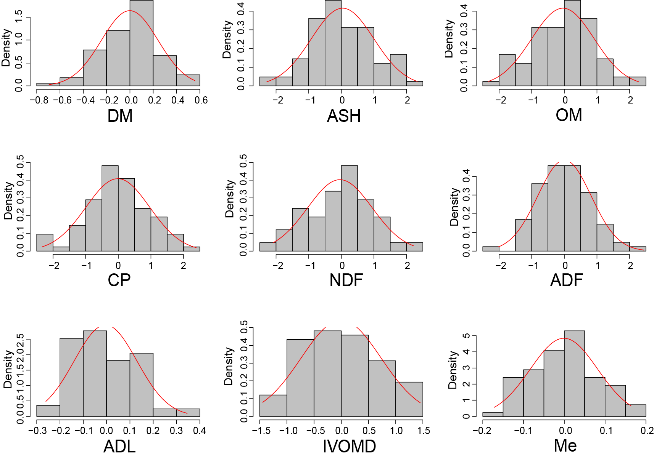

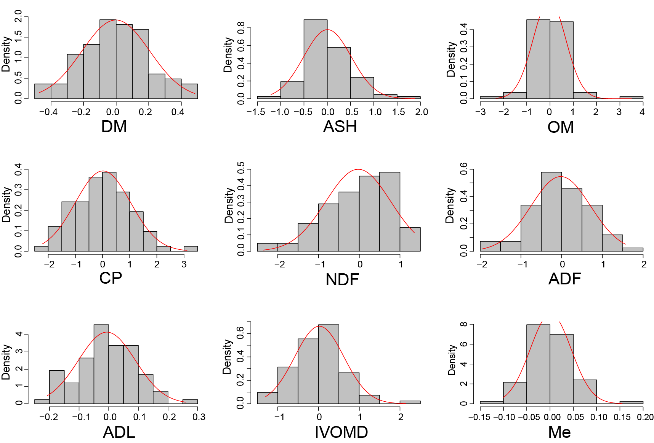


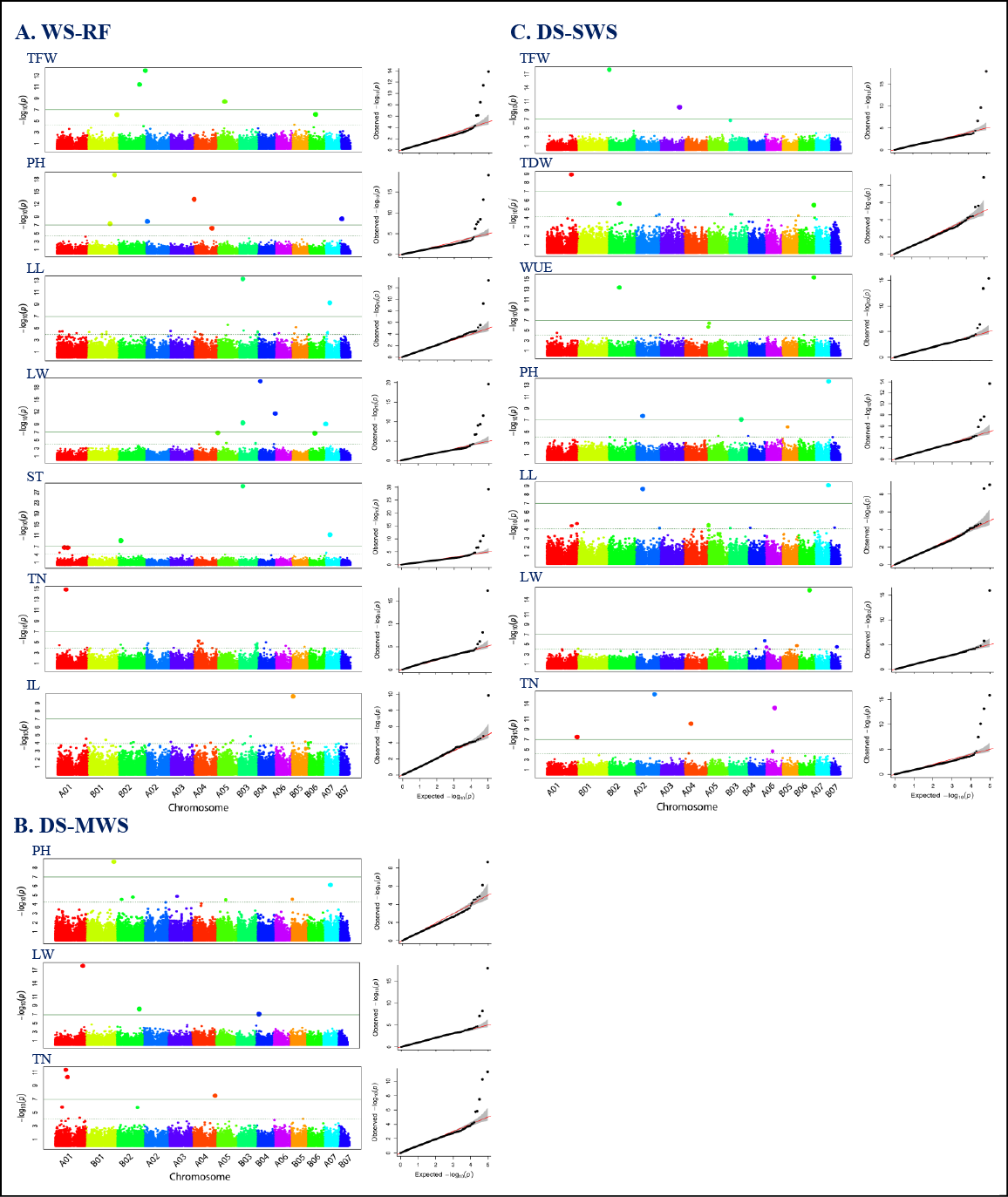


**Supplementary Figure 8.** Manhattan and quantile-quantile (Q-Q) plots showing the association of markers with the agronomic and morphological traits in wet season under rainfed (WS-RF) (A), dry season under moderate water stress (DS-MWS) (B), and dry season under severe water stress (DS-SWS) (C) conditions. Markers are on the x-axis per the 14-assembled chromosome (AC). The -log10 of P values are plotted on the y-axis. The traits are shown at the left, total fresh weight (TFW), total dry weight (TDW), water-use efficiency (WUE), plant height (PH), leaf length (LL), leaf width (LW), stem thickness (ST), internode length (IL), and tiller number (TN).


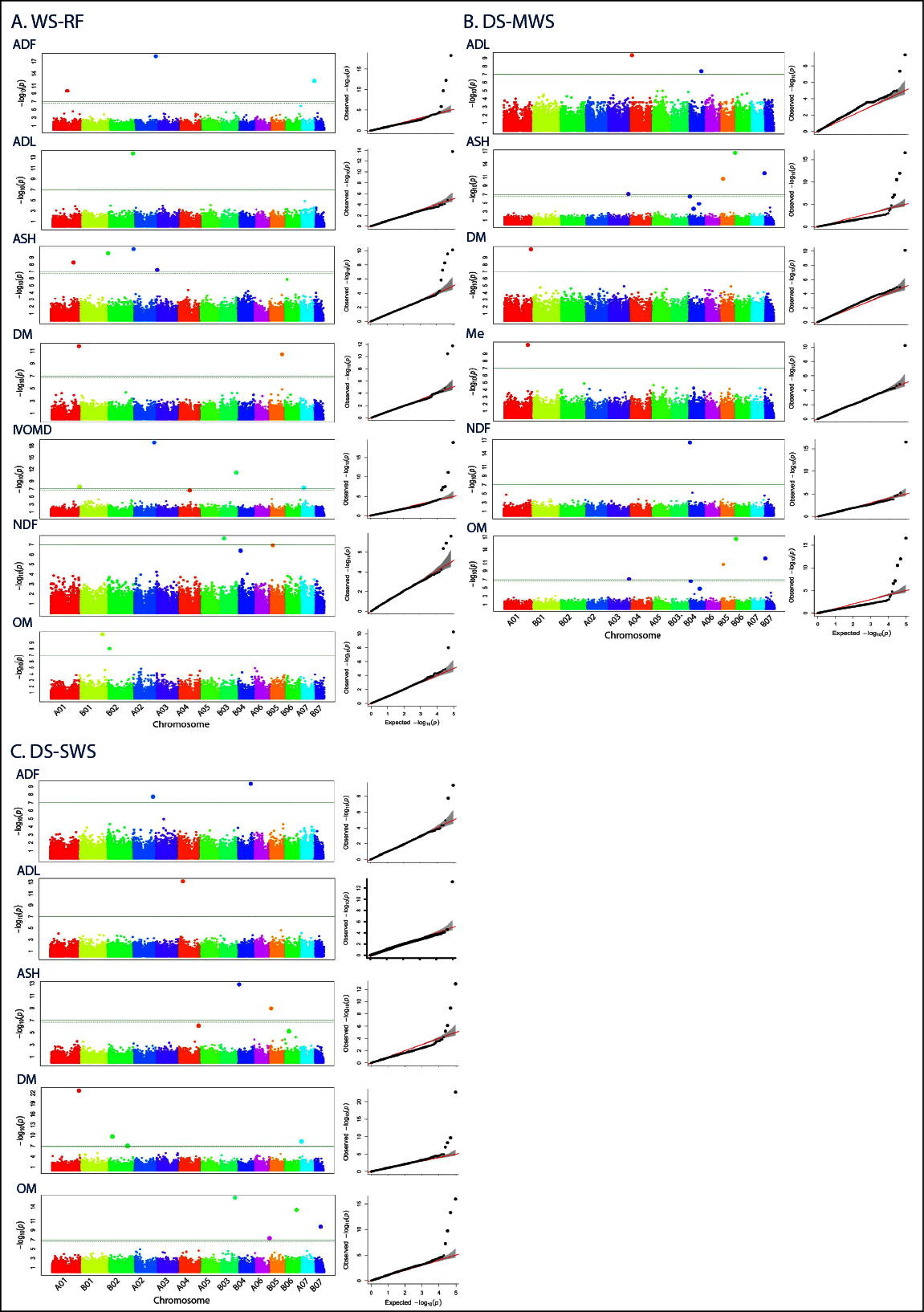


**Supplementary Figure 9.** Manhattan and quantile-quantile (Q-Q) plots showing the association of markers with the nutritional quality traits in wet season under rainfed (WS-RF) (A), dry season under moderate water stress (DS-MWS) (B), and dry season under severe water stress (DS-SWS) (C) conditions. Markers are on the x-axis per the 14-assembled chromosome (AC). The -log10 of P values are plotted on the y-axis. The traits are shown at the left: acid detergent fibre (ADF), acid detergent lignin (ADL), ash (ASH), dry matter (DM), metabolizable energy (Me), in vitro organic matter digestibility (IVOMD), neutral detergent fibre (NDF), organic matter (OM).

**Supplementary Figure 10**. A dendrogram showing clusters of the 83 Napier grass genotypes used in the genome-wide association study (GWAS). The cluster analysis was done by using selected 1000 robust SNP markers, which were distributed across the 14 assembled chromosomes (AC) of the Napier grass draft genome. The markers were highly polymorphic and independent from each other (pruned for LD at *r*^2^ = 0.5).
